# Organoid models of fibrolamellar carcinoma mutations reveal hepatocyte transdifferentiation through cooperative BAP1 and PRKAR2A loss

**DOI:** 10.1038/s41467-023-37951-6

**Published:** 2023-05-03

**Authors:** Laura Rüland, Francesco Andreatta, Simone Massalini, Susana Chuva de Sousa Lopes, Hans Clevers, Delilah Hendriks, Benedetta Artegiani

**Affiliations:** 1 The Princess Maxima Center for Pediatric Oncology, Utrecht, The Netherlands; 2 Department of Anatomy and Embryology, Leiden University Medical Center, Leiden, The Netherlands; 3 Hubrecht Institute, Royal Netherlands Academy of Arts and Sciences, Utrecht, The Netherlands; 4 Oncode Institute, Utrecht, The Netherlands; 5 University Medical Center Utrecht, Utrecht, The Netherlands; 6 Present Address: Pharma, Research and Early Development (pRED) of F. Hoffmann-La Roche Ltd, Basel, Switzerland

**Keywords:** Mechanisms of disease, Paediatric cancer, Genetic engineering

## Abstract

Fibrolamellar carcinoma (FLC) is a lethal primary liver cancer, affecting young patients in absence of chronic liver disease. Molecular understanding of FLC tumorigenesis is limited, partly due to the scarcity of experimental models. Here, we CRISPR-engineer human hepatocyte organoids to recreate different FLC backgrounds, including the predominant genetic alteration, the *DNAJB1-PRKACA* fusion, as well as a recently reported background of FLC-like tumors, encompassing inactivating mutations of *BAP1* and *PRKAR2A*. Phenotypic characterizations and comparisons with primary FLC tumor samples revealed mutant organoid-tumor similarities. All FLC mutations caused hepatocyte dedifferentiation, yet only combined loss of *BAP1* and *PRKAR2A* resulted in hepatocyte transdifferentiation into liver ductal/progenitor-like cells that could exclusively grow in a ductal cell environment. *BAP1*-mutant hepatocytes represent primed cells attempting to proliferate in this cAMP-stimulating environment, but require concomitant *PRKAR2A* loss to overcome cell cycle arrest. In all analyses, *DNAJB1-PRKACA*^*fus*^ organoids presented with milder phenotypes, suggesting differences between FLC genetic backgrounds, or for example the need for additional mutations, interactions with niche cells, or a different cell-of-origin. These engineered human organoid models facilitate the study of FLC.

## Abstract (web summary)

An in-depth understanding of the molecular pathogenesis of fibrolamellar carcinoma (FLC) is hampered due to limited human preclinical models. Here the authors engineer human organoids to reflect different FLC genetic backgrounds and show that hepatocytes can be a cell-of-origin of FLC that transdifferentiate into ductal/progenitor like cells due to combined BAP1 and PRKAR2A loss.

## Introduction

Fibrolamellar carcinoma (FLC) is a rare, lethal primary liver cancer. Effective therapies for FLC remain elusive, as FLC tumors infrequently respond to both chemotherapy and targeted therapies approved for hepatocellular carcinoma (HCC), highlighting its distinct biology^1,2^. It distinguishes itself from other primary liver cancers by its peculiar target group, *i.e*. adolescents and young adults, as well as its occurrence in absence of pre-existing chronic liver disease^1,2^. In addition to these specific clinical features, FLC is also characterized by its peculiar histology, *i.e.* lamellar bands^3,4^. Over the years, genomic analysis efforts have shed light on the genetic alterations linked to this clinically and histologically distinct liver cancer type. In 2014, the presence of a chromosomal rearrangement on chromosome 19 was described, which generates an in-frame chimeric fusion between the *DNAJB1* and *PRKACA* genes, and concomitantly poses an intergenic deletion of ca. 400 kb^5,6^. Typically, exon 1 or less often exon 2, of *DNAJB1* is fused to exons 2-10 of *PRKACA*, which keeps the N-terminal region of the heatshock protein DnaJ intact, as well as most of the kinase domain of the catalytic subunit α of protein kinase A (PKA)^5^. This fusion event is the most recurrent genetic alteration observed in FLC. Rarely, FLC has also been diagnosed in patients suffering from Carney complex^7^, caused by rare germline *PRKAR1A* mutations^8^.

Mixed FLC-HCC tumors, sharing features of both fibrolamellar and hepatocellular carcinoma, have also been described, yet only a subset harbors the chimeric fusion event^9,10^. Recently, a genetic background was linked to these non-fusion fibrolamellar-like tumors^11^. These carried genetic alterations related to PKA signaling, *i.e*. inactivating mutations in *PRKAR2A* (encoding the inhibitory regulatory subunit of PKA) and/or a chromosomal gain of *PRKACA*^11^, but importantly all combined with concomitant inactivating mutations of *BAP1*, an important tumor suppressor^12–14^, which is often found mutated in cholangiocarcinoma (CCA)^15^. How loss of *BAP1* contributes to the development of FLC-like phenotypes is currently unknown. Collectively, it appears that PKA-related mutations are an important genetic alteration occurring in tumors with FLC-like features. How these different genetic backgrounds affect liver cells and can drive the development of the same type of tumor, and how they molecularly relate or differ and may influence therapy is an unexplored topic.

FLC tumors typically display immunohistochemical evidence of both differentiated biliary and hepatocyte features^16,17^ and consequently the tumor-initiating cell in FLC is a debated issue^18^. The rare incidence of FLC complicates unravelling the molecular underpinnings of this liver tumor subtype. Overexpression of the *DNAJB1-PRKACA*^*fus*^ or endogenous recreation of the fusion by CRISPR-Cas9 could initiate liver tumor development in mice^19,20^, providing indications that the hepatocyte could constitute the cell-of-origin, but tumor latency was long. Instead, human-relevant models remain scarce. Patient-derived xenograft (PDX) models have allowed to long-term propagate human FLC tissue and to evaluate drug sensitivity^21–23^. Transcriptomic profiling of a PDX line displayed strong resemblance with biliary tree stem cells, and thus a progenitor cell capable of dual-lineage differentiation was instead proposed as putative cell-of-origin^21^. A recent study reported the establishment of 3D organoid cultures from primary and metastatic FLC tissues harboring the *DNAJB1-PRKACA*^*fus*^ background^24^. Relevant human models to address the impact of FLC mutations on initially healthy liver cells to probe the molecular and phenotypic consequences would help understanding the process and requirements of FLC tumorigenesis.

Human hepatocyte 3D organoid cultures represent long-term stable and expanding cultures of human fetal hepatocytes^25,26^ that can be robustly genome-engineered^26,27^, allowing to build liver disease models, such as for nonalcoholic fatty liver disease^28^. Here, we generate a set of human hepatocyte organoid models engineered to reflect different genetic backgrounds as identified in FLC or FLC-like cases. Using different CRISPR-Cas genome engineering strategies^29^, we recreate different genetic backgrounds (*DNAJB1-PRKACA*^*fus*^ and single or double *BAP1*^*KO*^*/PRKAR2A*^*KO*^ mutants) in order to unravel the molecular and phenotypic consequences of these genetic alterations from a hepatocyte point-of-view.

## Results

### CRISPR-engineered human hepatocyte organoid models reflecting different FLC mutational backgrounds

**Fig. 1:**
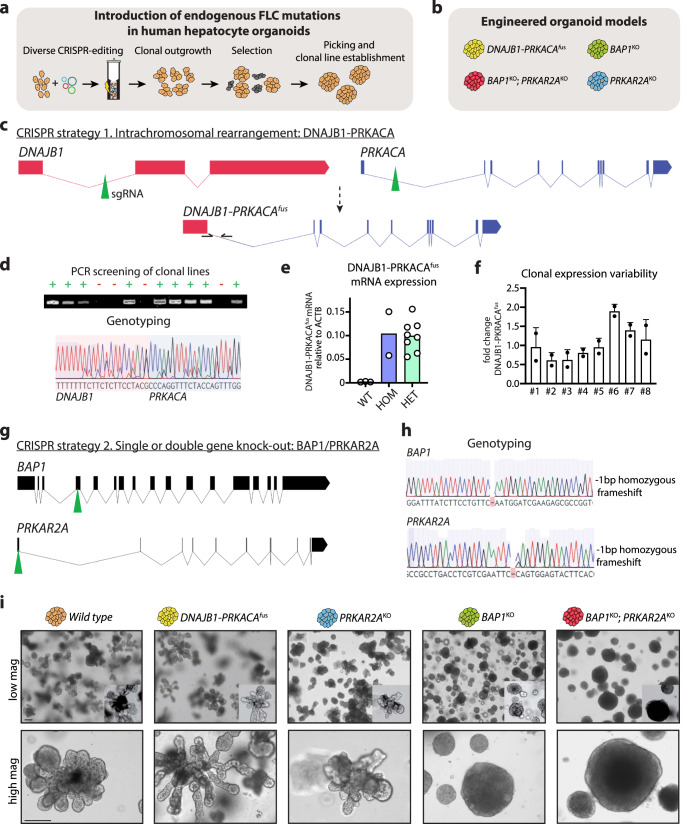
Generation of human hepatocyte organoid models reflecting different FLC mutations. **a**, Schematic of the strategy used to introduce the different FLC mutations using diverse CRISPR-editing. **b**, Overview of the different human hepatocyte FLC-mutant organoids generated in this study. **c**, Schematic of CRISPR strategy 1 to generate the *DNAJB1-PRKACA* fusion at the endogenous genetic loci. **d**, Representative results of PCR-screening and genotyping of outgrowing putative *DNAJB1-PRKACA*^*fus*^ mutant organoid lines (left). The presence of a band indicates that an amplicon has been generated using primers spanning the chimeric fusion (see **c**). Representative example of Sanger sequencing showing the correct sequence identity of the amplified genomic area as the chimeric *DNAJB1-PRKACA*^*fus*^ gene product (right). **e**, Bar plot showing mRNA expression of the chimeric *DNAJB1-PRKACA*^*fus*^ in wild type, heterozygous (HET) *DNAJB1-PRKACA*^*fus*^ and homozygous (HOM) *DNAJB1-PRKACA*^*fus*^ organoids, as assessed by qPCR analysis. Note its absence in wild type organoids. Each dot represents quantification in a different clonal line. Mean is plotted. *n* = 3, 8 and 2 for wild type, HET and HOM, respectively. **f**, Bar plot showing the mRNA expression variability of *DNAJB1-PRKACA*^*fus*^ across different clonal lines as assessed by qPCR. The average expression across all clonal lines was calculated, and the fold change of each individual line (each bar is a line) from the average is depicted. Each dot represents the quantification in the same line at 2 different culturing times, showing stable expression. Mean ± SD is plotted. **g**, Schematic of CRISPR strategy 2 to introduce *BAP1*^*KO*^ and/or *PRKAR2A*^*KO*^ mutations by harnessing NHEJ-mediated frameshift events. **h**, Representative examples of Sanger sequencing from PCR-based genotyping showing mutations in *BAP1* and *PRKAR2A*. **i**, Representative brightfield images at lower (top) and higher (bottom) magnification of the different mutant organoid lines that have been generated. Note the morphological differences observed in the *BAP1*^*KO*^ and *BAP1*^*KO*^;*PRKAR2A*^*KO*^ lines. Scale bar = 200 μm (low mag) and 75 μm (high mag). **d**, **i**, Representative of *n* = 3 and 4 independent experiments, respectively. Source data are provided as a Source Data file.

To study and compare the effects of the introduction of different genetic backgrounds found in FLC (*DNAJB1-PRKACA*^*fus*^) or FLC-like tumors (*BAP1*^*KO*^ and *PRKAR2A*^*KO*^) (from now on referred to as FLC mutations) in human hepatocytes, we employed tissue-derived human fetal hepatocyte organoids^25^. We used our previously optimized CRISPR-engineering approach^26^ to generate clonal human hepatocyte organoid lines carrying the different FLC mutations (Fig. 1a-b). To recreate the endogenous *DNAJB1-PRKACA* fusion, we employed a double sgRNA cut approach. While one sgRNA cuts in the intron between exon 1 and exon 2 of *DNAJB1*, the other cuts in the intron between exon 1 and exon 2 of *PRKACA*, thereby deleting the ca. 400 kb intergenic region (Fig. 1c). This type of approach is favored over the overexpression of the chimeric protein since it allows to maintain the endogenous regulatory sequences. Single cell-dissociated organoids were transfected with constructs carrying the DNAJB1 and PRKACA sgRNAs (two different sets of sgRNA were used in independent experiments), with a Cas9-expressing construct, and a *piggyBac*-based hygromycin resistance cassette for selection of transfected cells. This yielded the outgrowth of multiple organoids after hygromycin selection, that were individually expanded into lines. Several clonal lines were tested for the presence of the chimeric fusion, first by PCR using primers flanking the fusion point (Fig. 1c). Chromosomal engineering in human colon organoids using CRISPR-Cas9 was reported to be possible but with low efficiency, requiring disruption of TP53^30^. Instead, we observed a high efficiency in generating the *DNAJB1-PRKACA* chromosomal rearrangement, as for most of the transfected lines we detected the expected amplicon, and its identity was confirmed by Sanger sequencing (Fig. 1d), suggesting that these loci might be prone to genomic rearrangements. We tested whether this intrachromosomal deletion would translate into the expression of the chimeric fusion by qPCR. While, as expected, no amplicon was detected in wild type organoids, the *DNAJB1-PRKACA*^*fus*^ mRNA was detected in all engineered lines (Fig. 1e). The expression of the *DNAJB1-PRKACA*^*fus*^ across multiple clones was largely similar, yielding difference of ±2 fold at most (Fig. 1f). We could distinguish *DNAJB1-PRKACA*^*fus*^ heterozygous and homozygous lines by PCR-based genotyping (data not shown), but this difference in genotype did not translate into changes in fusion mRNA expression levels (Fig. 1e).

Next, we set to generate organoid lines carrying inactivating mutations in both *PRKAR2A* and *BAP1*, reflecting the second genetic subtype of FLC-like tumors, relying on the introduction of non-homologous end joining (NHEJ)-mediated frameshift events occurring upon Cas9 cleavage (Fig. 1g). To be able to address their potential functional cooperation, we also generated single mutant lines for both genes (Fig. 1b). Single cell-dissociated organoids were transfected with either PRKAR2A sgRNA, BAP1 sgRNA, or both, together with a Cas9-expressing construct as well as the *piggyBac*-based hygromycin resistance selection cassette. Also in this case, outgrowing organoids surviving after selection were expanded into lines and genotyped by PCR and Sanger sequencing, generating both single (*PRKAR2A*^*KO*^ or *BAP1*^*KO*^) and double mutant (*BAP1*^*KO*^*;PRKAR2A*^*KO*^) organoid lines (Fig. 1g-h). Altogether, we successfully generated a panel of human hepatocyte organoid lines reflecting the spectrum of endogenous mutations associated with different genetic backgrounds of FLC.

*DNAJB1-PRKACA*^*fus*^, *PRKAR2A*^*KO*^, *BAP1*^*KO*^, and *BAP1*^*KO*^*;PRKAR2A*^*KO*^ organoids were all viable and could be expanded in a similar manner as wild type organoids (Fig. 1i). We observed various degrees of phenotypic differences in these mutant cultures. Wild type human hepatocyte organoids usually display a typical cauliflower appearance^25,26^, which was more pronounced in the *DNAJB1-PRKACA*^*fus*^ organoids (Fig. 1i), forming longer tubular structures. On the contrary, *BAP1*^*KO*^ and *BAP1*^*KO*^*;PRKAR2A*^*KO*^ organoids appeared more round, dense, and dark (Fig. 1i).

### FLC mutations induce different degrees of hepatocyte phenotypic changes

**Fig. 2:**
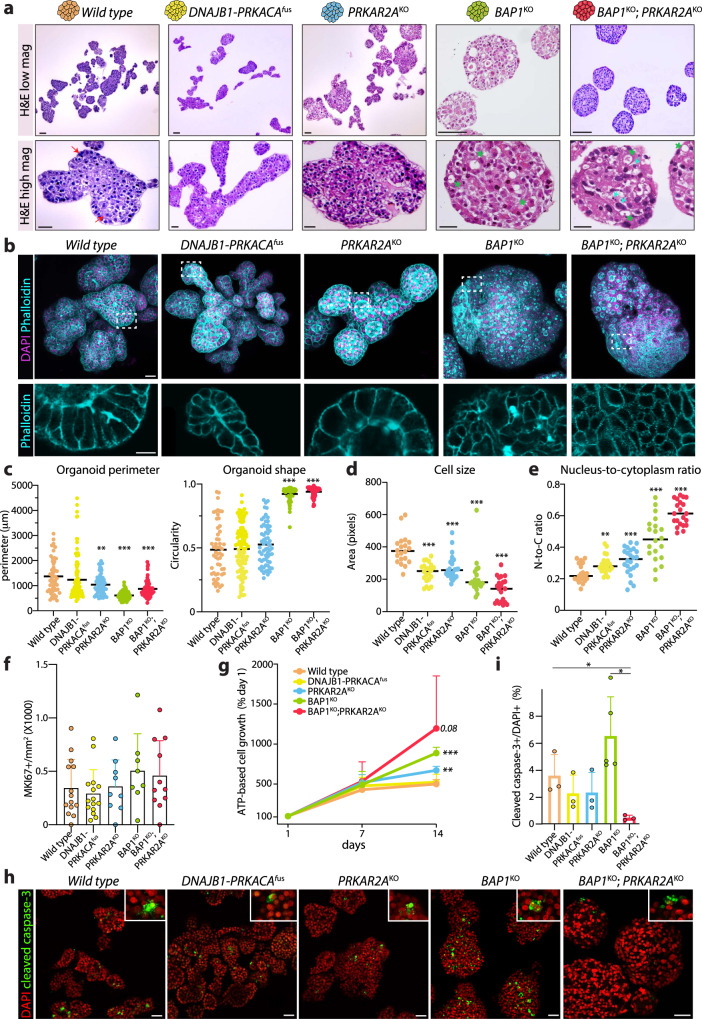
Cellular alterations in the FLC-mutant hepatocyte organoid models. **a**, Representative images of H&E staining at lower (top) and higher (bottom) magnification of the different FLC mutants and wild type organoids. Red arrows: polyploid cells, cyan stars: pale bodies, green arrows: nuclear pleomorphisms. See also Supplementary Fig. 1a. **b**, Representative images of the indicated staining in the different mutants. Representative of *n* = 2 independent experiments. **c**, Quantification of organoid perimeter (left) and shape (right) in the different mutants. Each dot represents a different organoid. Black line indicates the mean. *n* = 3 independent lines per genotype. **P < 0.01, ***P < 0.001 *versus* wild type; two-tailed Student’s *t*-test. **d**, Quantification of cell size in the different mutants. Each dot represents the size of a different cell. *n* = 3 independent lines per genotype. Black line indicates the mean. ***P < 0.001 *versus* wild type; two-tailed Student’s *t*-test. **e**, Quantification of the nucleus-to-cytoplasm ratio in the different mutants. Each dot represents quantification in a different cell. Black line indicates the mean. *n* = 3 independent lines per genotype. **P < 0.01, ***P < 0.001 *versus* wild type; two-tailed Student’s *t*-test. **f**, Bar plot showing quantification of the number of MKI67 + cells over organoid area. Each dot represents quantification in a different organoid. *n* = 3 independent lines per genotype. Mean ± SD is plotted. All mutants were not significant *versus* wild type; two-tailed Student’s *t*-test. See also Supplementary Fig. 1b. **g**, Line plot showing the acute growth following single cell dissociation of the different mutants over a period of 14 days as assessed by ATP assay. Data are shown as growth percentage as compared to day 1. Mean ± SD is plotted. **P < 0.01, ***P < 0.001 *versus* wild type; two-tailed Student’s *t*-test. **h**, Representative images of cleaved caspase-3 staining of the different mutant organoids. **i**, Bar plot showing the percentage of cleaved caspase-3+ cells over total nuclei (DAPI+). Representative of *n* = 3 independent lines per genotype. Mean ± SD is plotted. *P < 0.05 *versus* wild type; two-tailed Student’s *t*-test. Scale bars, 150 μm (low mag), 20 μm (high mag) (**a**), 30 μm (low mag), 10 μm (high mag) (**b**) and 50 μm (**h**). Exact sample size and exact *P* values for **c-g** and **i** are provided in the source data. Source data are provided as a Source Data file.

Prompted by our initial gross morphological observations, we performed histological examination to better understand the cellular consequences of the different FLC mutations. In wild type, *DNAJB1-PRKACA*^*fus*^, and *PRKAR2A*^*KO*^ organoids, cells displayed a polygonal and regular shape, typical of hepatocytes (Fig. 2a-b). Hematoxylin and eosin (H&E) staining highlighted instead in both *BAP1*^*KO*^ and *BAP1*^*KO*^*;PRKAR2A*^*KO*^ organoids multiple features of cellular dysplasia, particularly evident in the double mutant. Cells had irregular and very diverse shapes, often elongated, with a high degree of nuclear pleomorphism, including very large nuclei (Fig. 2a). Polyploid hepatocytes, a typical feature of the liver, were not often found in those mutants. Cellular inclusions, resembling pale bodies, often present in tumor FLC samples, were frequently detected in *BAP1*^*KO*^ and *BAP1*^*KO*^*;PRKAR2A*^*KO*^ cells (Fig. 2a and Supplementary Fig. 1a). Fibrous stromal bands, a typical histological feature of FLC^3,31^, were not observed in the *DNAJB1-PRKACA*^*fus*^ or the other mutant organoids. This may be explained by the pure hepatocyte nature of the organoids^25^ and therefore the absence of stromal cells. Whole organoid membrane phalloidin staining, revealed a drastically different cellular organization between the various mutant lines. In wild type organoids, hepatocytes were regularly organized into acinar structures, displayed a clear polarity, and formed a common internal canalicular system, as originally reported for these organoids^25^ (Fig. 2b). This peculiar organization was largely maintained in *DNAJB1-PRKACA*^*fus*^ and *PRKAR2A*^*KO*^ organoids, while this was lost in *BAP1*^*KO*^ and even to a higher degree lost in *BAP1*^*KO*^*;PRKAR2A*^*KO*^. These organoids lost the highly polarized organization and presented a relatively multi-cystic appearance with several internal lumina (Fig. 2b). Moreover, within the same organoid, the organization was very inhomogeneous, with patches of elongated cells often found in proximity to multi-luminal areas (Fig. 2b). Measurements of organoid shape, confirmed the more circular appearance of the *BAP1*^*KO*^ and *BAP1*^*KO*^*;PRKAR2A*^*KO*^ mutants that also resulted in a smaller perimeter (Fig. 2c). On a cellular level, *BAP1*^*KO*^*;PRKAR2A*^*KO*^ hepatocytes were considerably smaller as compared to wild type hepatocytes (Fig. 2d), and displayed an increased nucleus-to-cytoplasm ratio (Fig. 2e), a typical feature of malignant liver cells^32^. A similar, but less drastic, trend was also evident in the other mutants (Fig. 2d-e). Altogether, FLC mutations induce various degrees of cellular and morphological changes upon introduction in human hepatocyte organoids, with the most severe alterations being associated with concomitant loss of BAP1 and PRKAR2A.

We next addressed the growth characteristics of these mutants. Quantification of cellular proliferation of wild type and mutant organoid lines by MKI67 positivity showed no significant differences between the different conditions in homeostasis (Fig. 2f, Supplementary Fig. 1b). However, acute measurements of cellular growth upon single cell dissociation by ATP-based viability assay over 14 days, highlighted that *BAP1*^*KO*^ and *BAP1*^*KO*^*;PRKAR2A*^*KO*^ mutants presented with a growth advantage over wild type cells (Fig. 2g). Finally, we measured apoptosis rates by staining for cleaved caspase-3 (Fig. 2h). The *DNAJB1-PRKACA*^*fus*^ and the *PRKAR2A*^*KO*^ mutants presented a low level of apoptosis similar to wild type (Fig. 2h-i). Instead, the *BAP1*^*KO*^ single mutants showed a trend towards an increase in apoptotic cells, in agreement with previous reports on BAP1 loss in the liver^33^. Interestingly, apoptosis was virtually absent in *BAP1*^*KO*^*;PRKAR2A*^*KO*^ organoids, thereby suggesting that the concomitant loss of *PRKAR2A* overrules the intrinsic pro-apoptotic effects upon sole *BAP1* loss (Fig. 2i). The two different genetic backgrounds of FLC share the commonality of mutations in genes related to PKA signaling. Previous studies showed that fusion-driven FLC retains PKA activity and is cAMP responsive^5,34,35^ and hints of increased PKA activity have been associated with BAP1-driven FLC^11^. To further characterize our mutant lines, we measured the level of phospo-CREB (pCREB) by immunostaining as a proxy to address PKA activity, both in baseline and upon cAMP stimulation via forskolin stimulation (72 hrs) (Supplementary Fig. 2a-c). Wild type organoids responded to cAMP stimulation by increasing the pCREB level by ca. 7-fold. The *DNAJB1-PRKACA*^*fus*^ mutants showed a trend towards higher pCREB level as compared to wild type both in baseline as well as upon forskolin induction. This effect was more pronounced in the single *PRKAR2A*^*KO*^. Unexpectedly, we also noted a higher baseline pCREB level in *BAP1*^*KO*^ organoids, while these showed only a mild response to forskolin (less than 2-fold). Finally, we observed the highest relative induction of pCREB level in the *BAP1*^*KO*^*;PRKAR2A*^*KO*^ organoids, which might highlight the cooperation of the two mutations in driving PKA activation.

### Transcriptomic profiling reveals distinct molecular alterations posed by different FLC mutations in hepatocytes

**Fig. 3:**
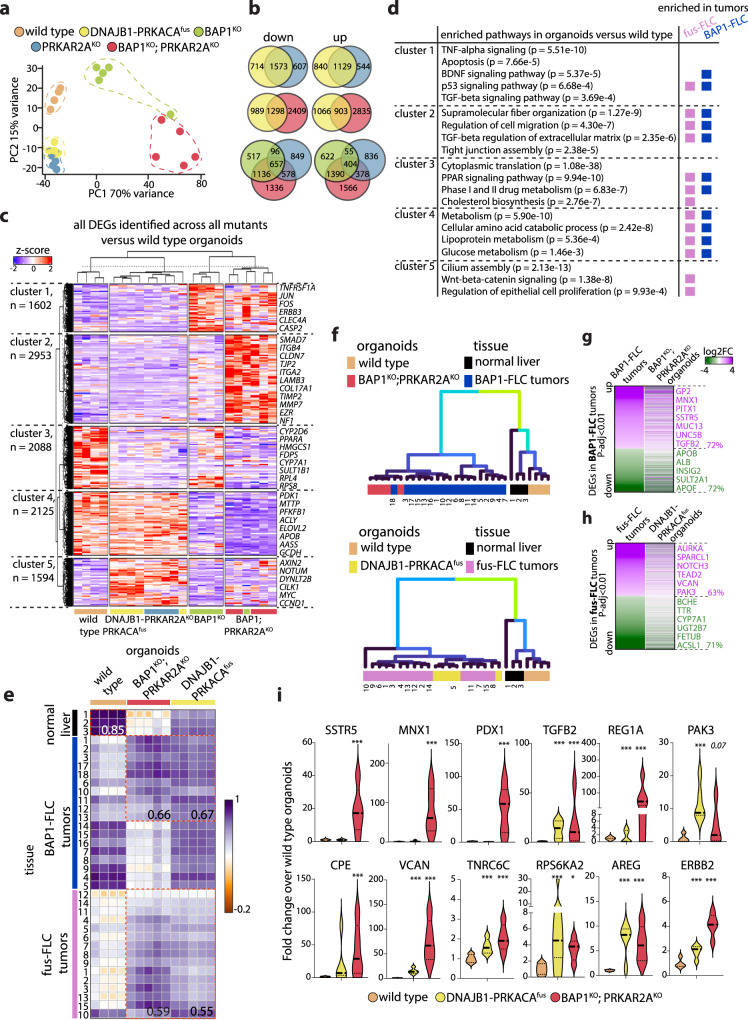
Molecular signatures of the FLC-mutant organoid models and tumor comparison. **a**, Principal component analysis (PCA) based on whole transcriptome analysis of wild type and the different engineered FLC-mutant organoid lines. Each dot represents the transcriptome of a different clonal line. **b**, Venn diagrams showing the (dis)similarities of the different mutant organoids based on the number of differentially expressed genes (downregulated and upregulated are shown separately) found in each mutant organoid model *versus* wild type (|log2FC| > 0.5 and *P-*adj < 0.05; Wald test; DESeq2). Color code indicating the different mutants as in **a**. See Supplementary Fig. 3. **c**, Heatmap depicting hierarchical sample clustering and gene clustering based on the expression of all differentially expressed genes found across all mutant organoid models *versus* wild type (|log2FC| > 0.5 and *P-*adj < 0.05; Wald test; DESeq2). Note the cluster consisting of *DNAJB1-PRKACA*^*fus*^ and single *PRKAR2A*^*KO*^ mutants. Each column is a different clonal line. **d**, GO-term analysis on the different gene clusters that emerged from hierarchical clustering of the differentially expressed genes (Fisher exact test; Enrichr). Comparisons are made with the GO-terms identified in both fusion-FLC and BAP1-FLC tumors (Hirsch et al.^11^). Boxes indicate that these pathways were also found significantly enriched in the respective FLC tumors *versus* normal livers. **e**, Correlation plot using whole transcriptomes comparing primary normal liver, fusion-FLC tumors, and BAP1-FLC tumors (Hirsch et al.^11^) with wild type, and engineered *DNAJB1-PRKACA*^*fus*^ and *BAP1*^*KO*^*;PRKAR2A*^*KO*^ organoid models. Relevant correlation coefficients are indicated. **f**, Hierarchical sample clustering comparing *DNAJB1-PRKACA*^*fus*^ organoids (left) and *BAP1*^*KO*^*;PRKAR2A*^*KO*^ (right) with the respective FLC tumor samples (Hirsch et al. ^11^). **g-h**, Comparison of the log2FC of differentially expressed genes (*P*-adj < 0.01, Wald test; DESeq2) identified in BAP1-FLC tumors (**g**) and fusion-FLC tumors (**h**) with the log2FC of these genes found in the respective engineered organoid models. The indicated percentages highlight the concordance in up- and down-regulated genes. Annotated genes in purple (upregulated) or green (downregulated) are examples of common significantly differentially expressed genes. **i**, Violin plots showing the fold change expression of reported upregulated FLC markers in *DNAJB1-PRKACA*^*fus*^ and *BAP1*^*KO*^*;PRKAR2A*^*KO*^ organoids relative to the expression in wild type organoids. *P-adj < 0.05, ***P-adj < 0.001 *versus* wild type (Wald test; DESeq2). Exact *P* values for **c** and **i** are provided in Supplementary Data 1. Source data are provided as a Source Data file.

To profile how these different FLC mutations affect hepatocytes, we performed whole transcriptomic analyses by bulk RNA-sequencing on all mutants and compared them with the expression profiles of wild type organoids. Principle component analysis (PCA) on whole transcriptomes revealed two distinct subgroups within the mutants (Fig. 3a). The first subgroup encompassed the single *PRKAR2A*^*KO*^ and the *DNAJB1-PRKACA*^*fus*^ organoids, suggesting a common PKA mutation-driven transcriptomic signature of these mutants. The other subgroup instead was determined by the presence of *BAP1* mutation. *BAP1*^*KO*^ organoid profiles were notably distinct from wild type organoids and with a much higher PC variance. The *BAP1*^*KO*^*;PRKAR2A*^*KO*^ organoids displayed the highest divergence from wild type organoids, further than the single *BAP1*^*KO*^ mutants on PC1, but also in the direction of *PRKAR2A*^*KO*^ and *DNAJB1-PRKACA*^*fus*^ on PC2 (Fig. 3a).

We performed differential gene expression analysis to describe the molecular changes by comparing each different mutant organoid model with the parental wild type organoids (|log2FC| > 0.5 and *P*-adj. < 0.05) (Supplementary Data 1). In general, we found much more transcriptomic changes in the *BAP1*^*KO*^*;PRKAR2A*^*KO*^ as compared to the other mutants. Pairwise comparison between *PRKAR2A*^*KO*^ and *DNAJB1-PRKACA*^*fus*^ revealed that a bulk of differentially expressed genes (1129 upregulated and 1573 downregulated) was shared between the two models, with an overall high transcriptomic correlation (Fig. 3b, Supplementary Fig. 3a-b). Comparison of *BAP1*^*KO*^*;PRKAR2A*^*KO*^ with both the single *BAP1*^*KO*^ and *PRKAR2A*^*KO*^ revealed a relatively small set of conserved differentially expressed genes between the three models (404 upregulated and 657 downregulated) (Fig. 3b, Supplementary Fig. 3a-b). *BAP1*^*KO*^*;PRKAR2A*^*KO*^ shared commonality with both single mutants (much more pronounced with *BAP1*). Interestingly, the double mutant presented with a large unique set of differentially expressed genes (1566 upregulated and 1336 downregulated). Altogether, this highlights a strong *BAP1* mutation-driven transcriptomic rewiring, but at the same time a unique transcriptomic effect upon simultaneous presence of the *PRKAR2A* mutation. Lastly, we compared the fusion organoids with the double mutant organoids, as these represent the two main currently known distinct genetic make-ups found in the FLC subtypes^5,11^. Despite being associated with histologically similar fibrolamellar tumors, the different set of mutations resulted into mutant hepatocyte organoids that were notably divergent on transcriptomic level, sharing 903 upregulated genes and 1298 downregulated genes (Fig. 3a-b, Supplementary Fig. 3a-b). *BAP1*^*KO*^*;PRKAR2A*^*KO*^ displayed the most extensive changes, in agreement with the histological characterization of these organoids (Fig. 2a-b).

Hierarchical clustering of all genes differentially expressed in at least one mutant *versus* wild type organoids confirmed separation of the single PKA-related mutants from the *BAP1*-related mutants (with the single *BAP1*^*KO*^ and double *BAP1*^*KO*^*;PRKAR2A*^*KO*^ still forming two subclusters) by different trends of gene expression (Fig. 3c). The hierarchical clustering on the differentially expressed genes revealed 5 distinct gene clusters (Fig. 3c). Cluster 1, 2, and 4 encompassed *BAP1* mutation-related gene expression changes, with cluster 2 being specific to the double mutant. Cluster 5 was confined to PKA mutation-related changes, while cluster 3 displayed a downregulation trend across all different mutants. We looked for enriched pathways in these clusters by gene ontology analysis and compared these with gene ontologies found by analysis of datasets on both fusion-FLC and BAP1-FLC tumor tissues^11,36^ (Fig. 3d, Supplementary Data 2). Cluster 3 included a set of overall downregulated genes, such as genes involved in drug metabolism (e.g*. CYP2D6, SULT1A2, FMO1, UGT2B4*), cholesterol biosynthesis (e.g*. HMGCS1, CYP7A1, FDPS, SQLE, DHCR7*) and PPAR signaling (e.g. *PPARA, PPARG*) (Fig. 3c-d, Supplementary Fig. 4a-b). Downregulation of genes encoding proteins involved in specific hepatocyte functions, such as cytochrome P450 enzymes, has also been observed in both FLC tumor types (Fig. 3d and refs. ^11,36,37^). We also observed downregulation of some genes related to protein translation (*i.e*. genes belonging to the ribosomal protein L (RPL) family) (Fig. 3c-d). Deregulation of ribosomal proteins has been associated with various cancers^38^. Genes in cluster 4 were instead mainly downregulated in the *BAP1*-single and/or double mutant organoids. This cluster included genes involved in amino acid catabolism (e.g. *GCDH, AHCY, QDPR*), lipid metabolism (e.g*. APOB, APOA2, PLIN2*), glycolysis (e.g. *PFKFB1/4, ENO1/3*), and triglyceride biosynthesis (e.g. *ACLY, ELOVL2/4/5/6, LPIN2*) (Fig. 3c-d, Supplementary Fig. 4a-b**)**. These changes might suggest a more pronounced impairment of liver-specific functions and deregulated metabolism in *BAP1*-mutated hepatocytes, with a tendency to worsen with additional *PRKAR2A* loss. Histological and clinical comparison of fusion-driven *versus* BAP1-driven FLC tumors indicated steatosis phenotypes specifically prominent in the BAP1-related tumors^11^. We therefore assessed steatosis levels in our mutant organoids by intracellular lipid staining. Intriguingly, this revealed a spontaneous mild lipid accumulation phenotype only in the single and double *BAP1*^*KO*^ organoid models (Supplementary Fig. 4c). Our transcriptomic data putatively suggest this may be the result of altered lipid metabolism gene expression, e.g. through the observed downregulation of the lipid export gene *APOB*^28^ only in *BAP1*-related mutants (Supplementary Fig. 4b).

Genes mostly upregulated in the single PKA mutants (cluster 5), encompassing both the fusion and the *PRKAR2A*^*KO*^, were mainly associated with the Wnt pathway, including *DKK1, DKK4, LEF1, AXIN2*, and *CCND1* (Fig. 3c-d, Supplementary Fig. 4a-b). Activating mutations in *CTNNB1* exacerbate and accelerate tumorigenesis in a fusion-driven FLC murine model^19^, and increased β-catenin protein expression and phosphorylation has been found in FLC even in absence of *CTNNB1* mutations^39^. Genes in cluster 1, typically upregulated in *BAP1*-mutated hepatocytes and more predominantly in single *BAP1*^*KO*^ mutants, were mostly related to apoptosis/stress response signaling (e.g*. JUN, FOS, CASP2, TRAF3/6*), TP53 (e.g. *ATF3, PLK3, P300, CREBBP*), and TGFβ (e.g. *TGFB1, AKT1, SMAD3*) signaling pathways (Fig. 3c-d, Supplementary Fig. 4a-b). BAP1 loss has been proposed to increase TGFβ signaling in pancreatic cancer^40^, and BAP1 has been linked to cell death and apoptosis^33,41,42^. The presence of *PRKAR2A*^*KO*^ could to a certain extent prevent these upregulations (Fig. 3c-d). Interestingly, some of these observations were corroborated by our functional data showing an increase in apoptosis level in *BAP1*^*KO*^, which is strongly reduced in *BAP1*^*KO*^*;PRKAR2A*^*KO*^ organoids (see Fig. 2h-i). Finally, amongst the pathways mostly upregulated in the double mutant (cluster 2), we noted genes involved in extracellular matrix organization and cellular junction components (e.g. *EZR, MMP7*, various collagens, and laminins), as well as genes involved in mucin secretion (e.g. *MUC1, MUC13*) (Fig. 3c-d, Supplementary Fig. 4a-b). Mucinous metaplasia is often observed in cancer, and various mucins are found upregulated in both subtypes of FLC^11,36^. Mucins are thought to be a critical determinant for cancer invasion and aggressiveness. Interestingly, most of the GO-terms found specifically in our clusters were also identified in transcriptomic datasets of fusion-FLC as well as BAP1-FLC tumors (Fig. 3d and^11,36,37^).

### Engineered FLC-mutant organoid models share transcriptomic similarities with FLC tumors

We then investigated how the transcriptomes of the engineered organoid models mimicking the two FLC genetic backgrounds (*i.e. DNAJB1-PRKACA*^*fus*^ and *BAP1*^*KO*^*;PRKAR2A*^*KO*^) compared with those from FLC tumor tissues. To this end, we used a published dataset on the full transcriptomes of a large cohort of both FLC genetic subtypes (Hirsch et al.^11^). We performed organoid-tumor comparisons by different means. First, we assessed similarities by performing correlation analysis based on whole transcriptomes (Fig. 3e). This showed a high correlation of the normal liver tissue with wild type organoids (correlation coefficient: 0.85) and a reduced similarity with the mutant organoids, in particular with *BAP1*^*KO*^*;PRKAR2A*^*KO*^. When focusing on the BAP1-FLC tumors, we noted a bimodal behavior. Over half of the tumors (*n* = 10) displayed a good correlation with both mutant organoid models (ca. 0.67), while showing a lower correlation with wild type organoids (Fig. 3e). These tumors also showed good correlation with fusion-FLC tumors (Supplementary Fig. 5a-b). Instead, the remainder of the BAP1-FLC tumors (*n* = 8) displayed a much stronger similarity with wild type organoids (Fig. 3e). Reassuringly, these same tumor samples also correlated highly to normal liver, suggesting a certain transcriptomic heterogeneity within these BAP1-FLC tumors (Supplementary Fig. 5a-b). Lastly, most of the FLC-fusion tumors showed good correlation with both the mutant organoid models (ca. 0.57) and lower correlation with wild type organoids (Fig. 3e). These similarities were corroborated by both gene set enrichment analysis (GSEA) as well as hierarchical clustering. GSEA using significantly downregulated or upregulated genes (|log2FC| > 0.5 and *P*-adj < 0.01, see Supplementary Data 3) identified in either fusion-FLC tumors or BAP1-FLC tumors (*versus* normal liver) revealed strong enrichment in the respective mutant organoid models (*versus* wild type organoids) (Supplementary Fig. 5c). Additionally, unsupervised hierarchical clustering showed that primary tumors clustered together with the respective organoid models, and were clearly distinct from normal liver tissue as well as wild type organoids (Fig. 3f). Accordingly, a gene signature using all differentially expressed genes identified in the mutant organoid models, was able to clearly separate the primary FLC tumor samples into 3 clusters: the BAP1-FLC tumors, the fusion-FLC tumors and the normal livers (Supplementary Fig. 5d). Vice versa, a “fibrolamellar signature” gene set defined by Lalazar et al.^23^ could be used to cluster our mutant organoid samples according to their mutational profiles (Supplementary Fig. 5e). Finally, when specifically looking into how the differentially expressed genes of the primary tumors are conserved in the organoid models, we identified a global, positive correlation for both models (Fig. 3g-h).

Based on a series of recent gene expression profiling efforts^5,10,11,17,36,37,43,44^, various markers typical of FLC tumors have been described^11,23,43,45^. Expression of genes related to EGF signaling have been recurrently found altered. Both *DNAJB1-PRKACA*^*fus*^ and *BAP1*^*KO*^;*PRKAR2A*^*KO*^ organoids also displayed such alterations, including upregulation of the EGF receptor HER2 (*ERBB2*) and the growth factor *AREG*. Importantly, FLC tumors show signs of neuroendocrine markers, especially the BAP1-FLC subtype^11^, and we found many of these induced, mostly in the *BAP1*^*KO*^*;PRKAR2A*^*KO*^ organoid model, including *GP2, SSTR5, CPE, PITX1*. Similarly, pancreatic progenitor markers were upregulated in both *DNAJB1-PRKACA*^*fus*^ and *BAP1*^*KO*^*;PRKAR2A*^*KO*^ organoids (such as *PAK3* and *REG1A*) or exclusively in the double mutant (such as *PDX1* and *MNX1*). Additionally, previously proposed markers specific for FLC tumors, *e.g. TNRC6C*, *RPS6KA2*, and *VCAN*, were significantly upregulated in both mutant models (Fig. 3i). Similarly, many genes found downregulated in fusion-FLC or BAP1-FLC tumors, were also consistently found downregulated in the engineered models (Fig. 3g-h, Supplementary Fig. 5f).

Our data show an overall resemblance of the mutant organoids with the relative FLC tumor transcriptomes, highlighting their value as a human physiological system to study the direct consequence and effects of FLC mutations on hepatocytes. However, not all expression changes identified in primary FLC tumors were mirrored in the engineered models. For instance, not all markers previously associated with fusion-FLC tumors were also altered in our engineered *DNAJB1-PRKACA*^*fus*^ or *BAP1*^*KO*^*;PRKAR2A*^*KO*^ organoids. These include, for example, *CA12* and *SLC16A14*. In addition, especially for the fusion organoid model, we observed -in general- a higher correlation with the downregulated genes than upregulated genes detected in primary fusion-FLC tumors (Fig. 3g-h). These findings are in agreement with the histological characterizations of the engineered organoids (Fig. 2), which revealed less drastic morphological aberrations in *DNAJB1-PRKACA*^*fus*^ organoids as compared to the double *BAP1*^*KO*^*;PRKAR2A*^*KO*^ mutants.

### FLC mutations reshape hepatocyte cell identity

Transcription factors control liver-specific functions and are fundamental to instruct and maintain cell identity and specification/differentiation during development and regeneration^46^. Several transcriptional programs were also affected by the introduction of the FLC mutations in human hepatocytes, particularly upon loss of both *BAP1* and *PRKAR2A* (Supplementary Fig. 6a). For instance, expression of *HNF4A*, considered the master regulator of hepatic differentiation, decreased in most mutants, while transcription factors such as *SOX4*, involved in hepatoblast proliferation and biliary cell specification^47^, were upregulated, overall suggesting ongoing changes in hepatic cell fate. These changes were most pronounced in the *BAP1*^*KO*^*;PRKAR2A*^*KO*^ organoids.

**Fig. 4:**
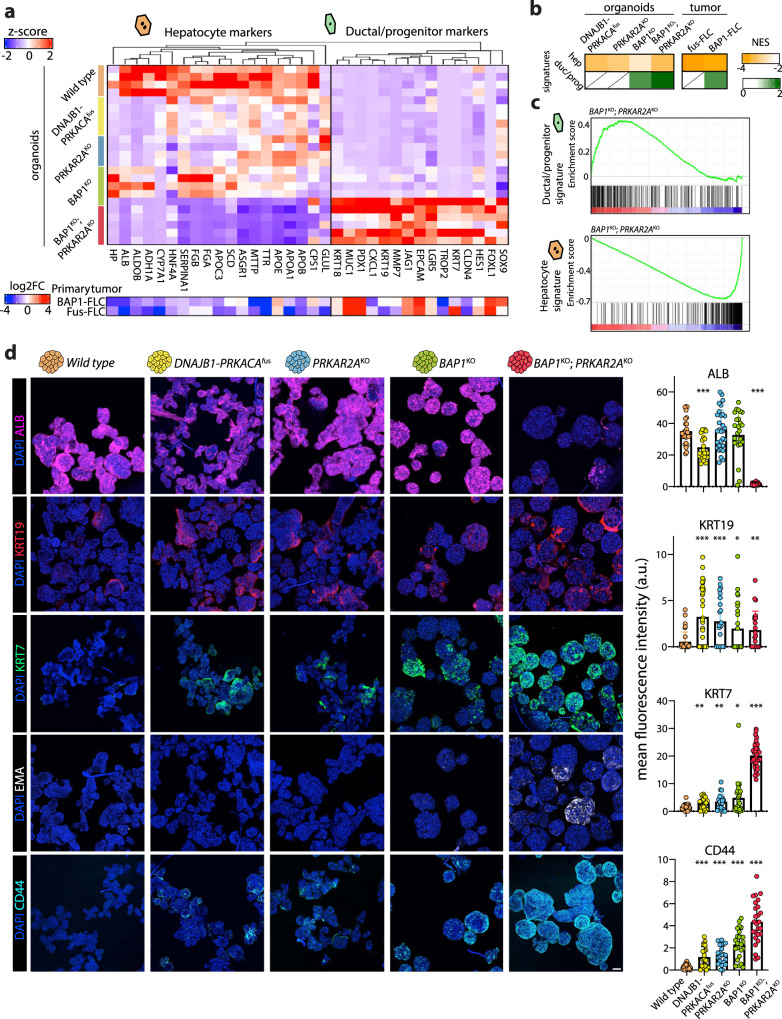
FLC mutations induce different degrees of cell identity alteration in human hepatocytes. **a**, Heatmap displaying the expression of selected hepatocyte and ductal/progenitor markers in the different FLC mutant organoids and wild type organoids. Note that *BAP1*^*KO*^*;PRKAR2A*^*KO*^ organoids display increased expression of ductal/progenitor markers. Each row is a different clonal line. Log2FC of the same markers in BAP1-FLC and fusion-FLC tumors *versus* normal liver (Hirsch et al. ^11^) is shown underneath. **b**, Normalized enrichment scores (NES) calculated for hepatocyte and ductal/progenitor signatures (derived from Aizarani et al.^49^, see Supplementary Data 3) assessed in the different mutant organoids compared to wild type organoids, as well as in BAP1-FLC and fusion-FLC tumors compared to normal liver (Hirsch et al. ^11^), based on GSEA. **c**, Enrichment plots corresponding to **b** for *BAP1*^*KO*^*;PRKAR2A*^*KO*^ organoids relative to wild type organoids for the hepatocyte and ductal progenitor signatures. **d**, Representative images of staining for hepatocyte (ALB), ductal/biliary (KRT7/19), and tumor (CD44 and EMA) markers across the different mutant organoids. Quantification for all markers is reported in the bar plots to the right (mean ± SD is plotted), except EMA as it is only expressed in *BAP1*^*KO*^*;PRKAR2A*^*KO*^ organoids. Each dot represents quantification in a different organoid. *n* = 3 independent lines per genotype and at least *n* = 4 organoids per line were quantified. *P < 0.05, **P < 0.01, ***P < 0.001 *versus* wild type; two-tailed Student’s *t*-test. The exact sample size and exact *P* values are provided in the source data. See Supplementary Fig. 6b. Scale bar = 100 μm. Source data are provided as a Source Data file.

Prompted by these observations, we performed a closer inspection into gene expression changes related to hepatic cell identity. Wild type hepatocyte organoids displayed strong expression of hepatocyte markers (e.g. *ALB, APOB, TTR, ASGR1*), while expression of biliary and progenitor markers was low (Fig. 4a). In both *DNAJB1-PRKACA*^*fus*^ and *PRKAR2A*^*KO*^ as well as the *BAP1*^*KO*^*;PRKAR2A*^*KO*^ mutant organoids, we observed a trend of decreased expression of most hepatocyte markers, which was most pronounced in the double mutant (Fig. 4a). Interestingly, a similar trend for many of these markers was also found back in both primary FLC tumors (Fig. 4a, and^11^). Instead, as a characteristic of the *BAP1*^*KO*^*;PRKAR2A*^*KO*^ organoids, we noted high expression of ductal/progenitor markers, which suggested a synergistic mutational effect that impacts on cellular identity (Fig. 4a). Amongst others, we noted increased expression of typical progenitor markers *EPCAM* and *LGR5*^48^, as well as the more recently described *TROP2 (TACTSD2)*^49^, a marker of bipotent liver progenitors (Fig. 4a). Markers typical of the biliary lineage^50^, including *KRT7*, *KRT19*, and *SOX9*, were accordingly also enriched. One *BAP1*^*KO*^ line out of the five lines analyzed displayed a similar transcriptomic behavior as the double mutant in this aspect, and its whole transcriptome neared the cluster of double mutants on PCA (Fig. 3a). This suggests a certain level of behavioral heterogeneity upon *BAP1* mutation, which could be due to its role as epigenetic regulator and in this regard, the cohort of BAP1 FLC-tumors also showed signs of transcriptomic heterogeneity (Supplementary Fig. 5a-b). Based on multiple typical hepatocyte (298 genes) and ductal/progenitor (219 genes) markers, cell identity signatures for these cell types were previously defined^49^. GSEA using these marker sets (see Supplementary Data 3) confirmed that all mutants displayed a negative enrichment of the hepatocyte signature (Fig. 4b-c). In contrast, a positive enrichment of the ductal/progenitor signature was obtained only in *BAP1*^*KO*^ and more strongly in *BAP1*^*KO*^*;PRKAR2A*^*KO*^ organoids (Fig. 4b-c). We also assessed these signatures in the primary FLC tumors. This revealed concordance between our mutant organoids and primary tumor tissues, which highlighted the enrichment of the ductal/progenitor signature only in BAP1-FLC tumors (Fig. 4b).

We then addressed protein expression changes in the mutant organoids by immunofluorescence staining for some of the typical markers of liver cell identity, which are also used for the characterization of primary FLC samples^16^ (Fig. 4d and Supplementary Fig. 6b). This confirmed decreased protein expression of ALB, a typical hepatocyte marker in *BAP1*^*KO*^;*PRKAR2A*^*KO*^ organoids (Fig. 4d). ALB expression was also reduced in *DNAJB1-PRKACA*^*fus*^ organoids but was still clearly present. As compared to HCC, FLC often stains positive for biliary and CCA markers, such as KRT7^16,51,52^. A certain percentage of FLC tumors stains also positive for KRT19^16^. Expression of both those markers was enriched in all mutant organoids, and especially KRT7 expression in the double mutants was abundant. In agreement with their more pronounced phenotype and acquisition of a ductal identity, the double mutants were also the only model showing expression of EMA (*MUC1*) (Fig. 4d and Supplementary Fig. 6b), another typical FLC tumor histological biliary marker^16^. Positive expression for CD44, a typical tumor marker strongly expressed in FLC^53,54^, was detected in all mutants -but not in wild type organoids- and again more strongly in the double mutant. Interestingly, not all organoids and not all the cells in the organoids stained positive for the different markers. Since the lines are clonally derived from single mutated hepatocytes, this suggests the natural acquisition of a certain level of phenotypic heterogeneity (Supplementary Fig. 6b). In conclusion, the different FLC mutations imposed a certain degree of dedifferentiation of the hepatocytes, while the concomitant inactivation of *BAP1* and *PRKAR2A* resulted in the most drastic cell fate switch towards a more liver ductal cell/progenitor-like phenotype.

### Selective growth of double *BAP1* and *PRKAR2A*-mutant hepatocytes in an environment stimulating liver ductal cell proliferation

**Fig. 5:**
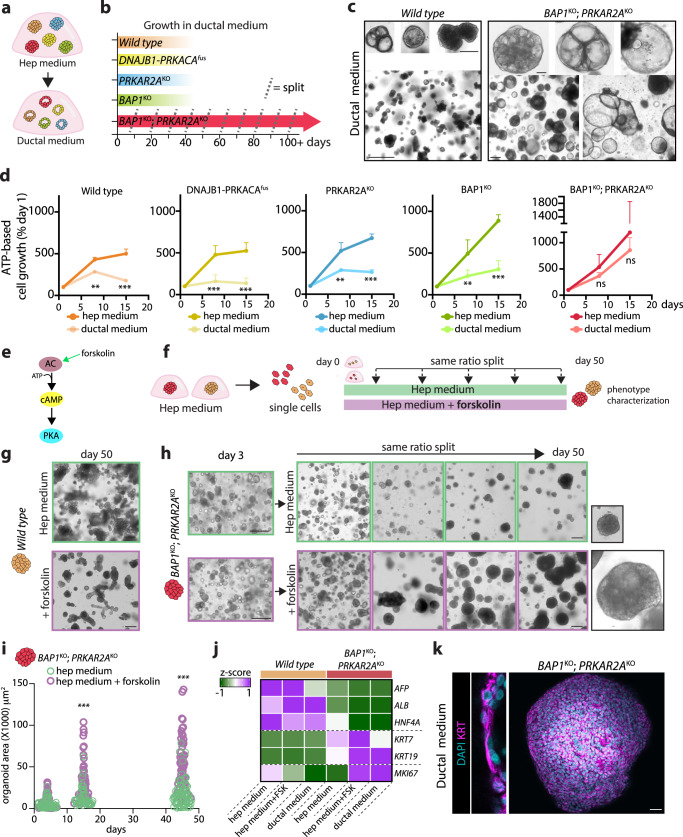
Double *BAP1*- and *PRKAR2A*-mutant hepatocyte organoids exclusively adapt and thrive in a ductal cell environment. **a**, Experimental set-up to test the capacity of the different organoid lines to grow in an environment stimulating ductal cell growth. **b**, Schematic representing the culture time under which the different organoid lines could be maintained in ductal medium. Dashed line indicates a splitting (expansion) event. Results are derived from observations in *n* = 3 independent lines per genotype. See Supplementary Fig. 7b. **c**, Representative brightfield images of (arrested) wild type organoids *versus* (growing) *BAP1*^*KO*^*;PRKAR2A*^*KO*^ organoids in ductal medium at 30 days post medium switching. **d**, Line plots showing the acute growth following single cell dissociation in hep or ductal medium of the different lines over 15 days as determined by ATP assay. Data represent growth percentage as compared to day 1. Mean ± SD is plotted. *n* = 2 independent replicates per genotype. **P < 0.01, ***P < 0.001 *versus* hep medium; two-tailed Student’s *t*-test. **e**, Schematic of forskolin-mediated PKA stimulation. **f**, Experimental set-up to evaluate the effect of forskolin on the growth of the different lines. **g**, Representative brightfield images of wild type organoids cultured from single cells in hep medium with or without forskolin supplementation at day 50. **h**, Representative brightfield images of *BAP1*^*KO*^*;PRKAR2A*^*KO*^ organoids cultured from single cells in hep medium with or without forskolin supplementation over 50 days. **i**, Quantification of the area of *BAP1*^*KO*^*;PRKAR2A*^*KO*^ organoids when cultured in hep medium with or without forskolin supplementation over 50 days. *n* = 65, 49 and 72 organoids for hep medium and *n* = 52, 46 and 49 organoids for hep medium + forskolin at day 4, 15, and 45, respectively. **P < 0.01, ***P < 0.001 *versus* hep medium; two-tailed Student’s *t*-test. **j**, qPCR analysis of expression of selected hepatocyte (*AFP, ALB, HNF4A*), ductal/progenitor (*KRT7, KRT19*), and proliferation (*MKI67*) markers in wild type and *BAP1*^*KO*^*;PRKAR2A*^*KO*^ organoids cultured in the indicated medium. n = 3 independent measurements per gene and condition. **k**, Representative image of KRT staining of a *BAP1*^*KO*^*;PRKAR2A*^*KO*^ organoid cultured in ductal medium. **c**, **g**, **h**, **k** Representative of *n* = 3, 3, 3 and 2 independent experiments, respectively. Scale bars, 200 μm (low mag) and 50 μm (high mag) (**c**), 200 μm (**g**, **h**) and 50 μm (**k**). Exact sample size and exact *P* values for **d** and **i** are provided in the source data. Source data are provided as a Source Data file.

Epithelial cells from the liver can be cultured and expanded as 3D organoids as two separate cultures: the liver ductal organoids arising from EpCAM^+^ cells^48,55^, and the ALB^+^ hepatocyte organoids^25,26^ used in the current study. Each of these cultures requires their own culture medium, resembling the in vivo niches, in order to sustain the expansion of the respective liver epithelial cells. Given our transcriptomic indications that the FLC mutations impact -to various degrees- on hepatocyte identity, we questioned whether any of these mutants (normally cultured in hepatocyte (hep) medium) could instead proliferate in culture medium normally supporting the expansion of human liver ductal cells (ductal medium) (Fig. 5a, Supplementary Fig. 7a). Full-size wild type organoids when switched to ductal medium could be long-term maintained but, importantly, not expanded in ductal medium (ca. 30 days) (Fig. 5b-c, Supplementary Fig. 7b), forming occasionally small internal lumens. The *DNAJB1-PRKACA*^*fus*^, *PRKAR2A*^*KO*^, and *BAP1*^*KO*^ mutants all responded similarly (Fig. 5b, Supplementary Fig. 7b). The *BAP1*^*KO*^*;PRKAR2A*^*KO*^ organoids instead thrived in this ductal cell-expanding medium. These organoids grew and could be repeatedly expanded long-term (Fig. 5b-c, Supplementary Fig. 7b). Of note, while the double mutants showed signs of slower growth after about 6-7 months of culturing in hep medium, this was instead not observed when they were cultured in ductal medium. Morphologically, the *BAP1*^*KO*^*;PRKAR2A*^*KO*^ organoids became more cystic in the ductal medium (Fig. 5c, Supplementary Fig. 7b), with some resembling the typical appearance of human liver ductal organoids^48^. An extent of morphological heterogeneity was present, ranging from more dense/multi-cystic to completely cystic organoids (Fig. 5c). These findings were further substantiated by the very limited single-cell outgrowth when switched to ductal medium of both wild type and all other mutant organoids (and therefore their incapacity to grow into lines), except the *BAP1*^*KO*^*;PRKAR2A*^*KO*^ organoids (Fig. 5d, Supplementary Fig. 7c-d). We followed up by staining the organoids for the proliferation marker MKI67 in ductal medium. Confirming our results on organoid growth, *BAP1*^*KO*^*;PRKAR2A*^*KO*^ organoids displayed MKI67 positivity, while the wild type, *DNAJB1-PRKACA*^*fus*^, and *PRKAR2A*^*KO*^ organoids displayed almost no MKI67^+^ cells (Supplementary Fig. 8a-b). Surprisingly, the single *BAP1*^*KO*^ mutant organoids still presented MKI67^+^ cells, even though they clearly did not expand in ductal medium (Supplementary Fig. 8a-b). The *BAP1*^*KO*^ organoids are histologically and molecularly the most similar to the double mutant (Figs. 2–4), and are indeed also enriched for the progenitor/ductal cell signature (Fig. 4b). However, this single mutation appeared not sufficient to fully convert the initially targeted hepatocytes into a transdifferentiated cell population able to expand in a ductal environment (Fig. 5d, Supplementary Fig. 7b, d). Altogether, this suggested that, due to a cooperation between *BAP1*^*KO*^ and *PRKAR2A*^*KO*^ mutations, human hepatocytes acquire a cellular plastic ground state, which allows them to not only respond to signaling promoting hepatocyte growth, but as well to signaling promoting liver ductal cell expansion. This is in obvious contrast with wild type hepatocytes and all other mutants which arrested their growth in ductal medium.

### Activation of cAMP drives growth of double *BAP1-* and *PRKAR2A*-mutant hepatocytes as ductal-like cells

The respective ductal and hepatocyte expansion media differ for some components (Supplementary Fig. 7a). Among those, the presence of forskolin in the ductal medium, which is an adenylyl cyclase (AC) activator, struck our attention, given that AC activation promotes cAMP production and eventually PKA activation^56^ (Fig. 5e). We thus wondered whether putatively enhanced PKA activation could contribute to the selective growth of the double mutants observed in response to the ductal environment. We evaluated the effect of PKA activation on the growth of single cells from wild type and mutant organoids when cultured and passaged in hep medium supplemented with forskolin (Fig. 5f). The presence of forskolin reduced (but to a lesser extent than ductal medium) the growth of wild type, *DNAJB1-PRKACA*^*fus*^, and *PRKAR2A*^*KO*^ organoids (Fig. 5g, Supplementary Fig. 9a). The growth of *BAP1*^*KO*^ organoids was instead not affected by the addition of forskolin (Supplementary Fig. 9a-c), which could be tentatively linked to our earlier observation that *BAP1*^*KO*^ organoids minimally increased pCREB levels in response to forskolin (Supplementary Fig. 2b-c). Instead, forskolin improved the long-term growth of *BAP1*^*KO*^*;PRKAR2A*^*KO*^ cells, that could generate multi-cystic organoids (Fig. 5h, Supplementary Fig. 9a). We quantified the organoid size, which confirmed that the presence of forskolin selectively enhanced the growth of the double mutant organoids (Fig. 5i, Supplementary Fig. 9a, c). Additionally, we corroborated our findings by withdrawal of forskolin from the ductal medium, which accordingly resulted in reduced growth and an absence of MKI67^+^ cells in double mutant organoids (Supplementary Fig. 8c-d).

Wild type organoids in both ductal medium and in hep medium plus forskolin largely preserved the expression of hepatocyte markers (e.g. *HNF4A, ALB*) and did not increase keratin expression, while *MKI67* expression gradually reduced (Fig. 5j). Instead, the double mutant further decreased hepatocyte markers and increased ductal and proliferation markers, in both ductal medium as well as simply in presence of forskolin (Fig. 5j). Protein staining highlighted strong pan-KRT expression and the ductal-like appearance of the double mutant organoids, with a monolayer epithelium constituted by small and rather flat cells (Fig. 5k, Supplementary Fig. 8e).

### *BAP1* mutation primes progression to the cell cycle in a ductal environment but requires *PRKAR2A* loss to overrule mitotic arrest

**Fig. 6:**
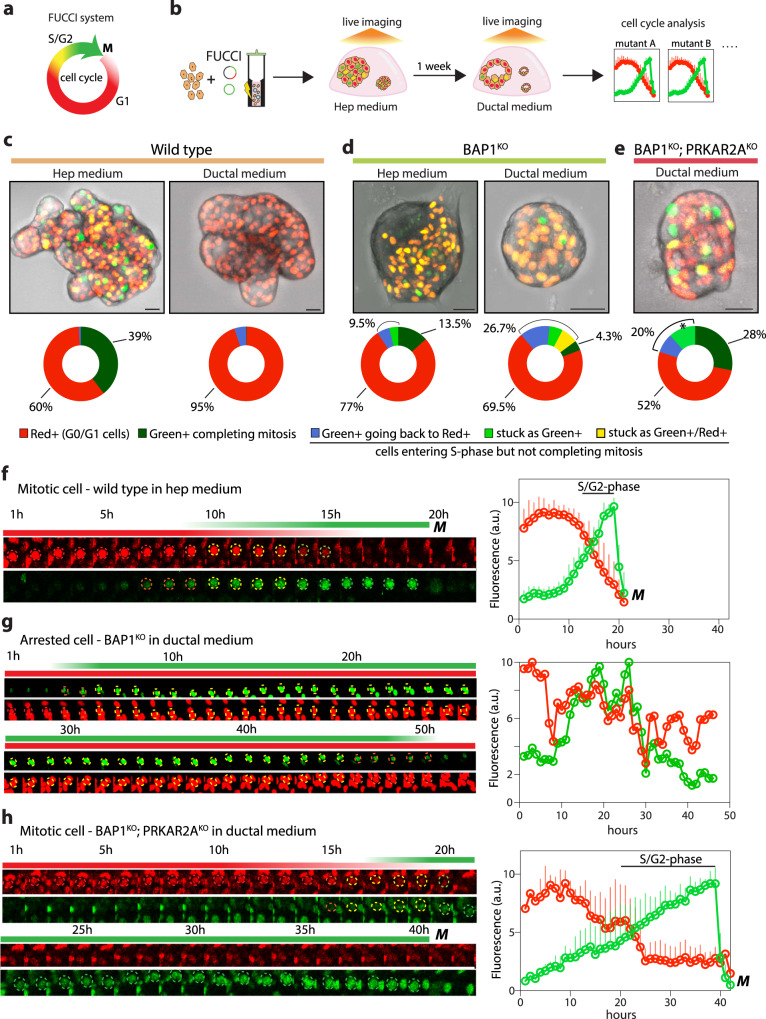
Concomitant loss of *PRKAR2A* in *BAP1*-mutant hepatocytes is required to complete mitosis in a ductal cell environment. **a**, Schematic of the FUCCI system used (Clover-Geminin (Green^+^)-IRES-mKO2-Cdt1 (Red^+^)). Cells in G0/G1 phase are marked red, while early S phase is marked yellow, and cells in S/G2/M phases are marked green. **b**, Workflow used to assess cell cycle dynamics. Cells are transfected with a *piggyBac*-transposable FUCCI cassette. FUCCI-labelled organoids are live-imaged in hep medium and/or ductal medium, and their cell cycle is analyzed. **c–e**, Top: Representative brightfield images overlaid with Green/Red fluorescence of FUCCI-labelled wild type (**c**), *BAP1*^*KO*^ (**d**), *BAP1*^*KO*^*;PRKAR2A*^*KO*^ (**e**) organoids in hep and ductal medium. Bottom: Pie charts indicating the percentages of cell cycle events in the different conditions over a ca. 72 h imaging session. Note that Green^+^ cells in *BAP1*^*KO*^*;PRKAR2A*^*KO*^ organoids most likely represent cells that progress to divide but fall outside the recorded time window. Amount of cells quantified: *n* = 828 for wild type in hep medium, *n* = 428 for wild type in ductal medium, *n* = 374 for *BAP1*^*KO*^ in hep medium, *n* = 171 for *BAP1*^*KO*^ in ductal medium, *n* = 295 for *BAP1*^*KO*^*;PRKAR2A*^*KO*^ in ductal medium. **f**, Example of a cell progressing through the cell cycle and completing mitosis in wild type organoids cultured in hep medium. On the right, an average trace of the Green/Red signal is shown. Mean ± SD is plotted. M = mitosis. *n* = 12 cells. **g**, Example of a cell arrested along the cell cycle in *BAP1*^*KO*^ organoids cultured in ductal medium. On the right, the trace of the Green/Red signal is shown. n = 1 cell. **h**, Example of a cell progressing through the cell cycle and completing mitosis in *BAP1*^*KO*^*;PRKAR2A*^*KO*^ organoids cultured in ductal medium. On the right, an average trace of the Green/Red signal is shown. Mean ± SD is plotted. M = mitosis. Note the prolonged S/G2 phase. *n* = 6 cells. **c-h**, All representative of *n* = 3 independent experiments. Scale bars, 25 μm (**c–e**). Source data are provided as a Source Data file.

Our results showed that the presence of concomitant mutations in *BAP1* and *PRKAR2A* is the only genetic background that allows the initially-targeted hepatocytes to proliferate in a ductal environment, presumably due to their transdifferentiation (Fig. 5b–d, Supplementary Fig. 7b, d). Under this condition however, *BAP1*^*KO*^ organoids were also enriched for MKI67^+^ cells (Supplementary Fig. 8a-b), despite their lack of expansion (Fig. 5b–d, Supplementary Fig. 7b, d). We therefore reasoned that analysis of cell cycle dynamics in these different mutants would help to understand possible synergistic actions of the two mutations. To this aim, we transfected wild type, *BAP1*^*KO*^;*PRKAR2A*^*KO*^ as well as *BAP1*^*KO*^ organoids with a *piggyBac*-based FUCCI plasmid system (Clover-Geminin(Green^+^)-IRES-mKO2-Cdt1(Red^+^))^57^ and performed live-imaging (ca. 60 hrs/session) to assess cell cycle kinetics (Fig. 6a-b). We first evaluated the cell cycle behavior of wild type hepatocytes in hep medium (Fig. 6c). Within the recorded timeframe and over the labelled population, we noted many mitotic events (ca. 40%). These cells showed a very regular cell cycle progress and cell cycle length, typically lasting ca. 20 h (Fig, 6f, Supplementary Fig. 10a-b). About 60% of the cells were single Red^+^, indicating those cells to reside in G0/G1 phase^57^. We then imaged wild type hepatocytes after they were maintained in ductal medium for a week (Fig. 6b). In this setting, we did not observe mitotic events and essentially all the hepatocytes remained Red^+^, indicating no cell cycle activity (Fig. 6c, Supplementary Fig. 10a-b).

We next analyzed *BAP1*^*KO*^-FUCCI organoids. In hep medium, these organoids could complete mitosis but at lower frequency than wild type hepatocytes (13.5%) and with a slightly increased cell cycle length, while 77% of the cells remained Red^+^ throughout the recorded timeframe (Fig. 6d, Supplementary Fig. 10a-b). In addition, we noted a substantial percentage of cells with aberrant behavior (9.5%): these cells were either stuck as Green^+^ cells for a prolonged time or reverted from initially being Green^+^ back to Red^+^, altogether presumably presenting arrested cells that entered S-phase but did not complete mitosis (Fig. 6d). We then analyzed their behavior in ductal medium. Slightly less cells remained Red^+^ (70%, *i.e*. in G0/G1 phase), and we noted a significant proportion of aberrant cells (26.5%) (Figs. 6d, g, Supplementary Fig. 10c). These cells were either stuck as Green^+^ (S-phase arrested), Green^+^/Red^+^ (early S-phase arrested), or reverted from Green^+^ back to Red^+^ (going back to G0/G1), but all not being able to complete division. These observations are in stark contrast with the behavior of wild type hepatocytes, where cells remained in G0/G1. We interpreted these findings as *BAP1*^*KO*^ hepatocytes being primed to proliferate in ductal medium, in line with the observed increase in MKI67 + cells (Supplementary Fig. 8a-b), but predominantly encountering cell cycle arrest (Fig. 6d, g). Instead, when recording the *BAP1*^*KO*^;*PRKAR2A*^*KO*^ mutant in ductal medium, we found many cells able to successfully progress towards division and undergo mitosis over time (28%), while only 52% remained in G0/G1 phase (Red^+^) (Figs. 6d, h, Supplementary Fig. 10a-b). We also analyzed their cell cycle kinetics, and noticed a much longer cell cycle, especially of the S phase, as compared to wild type proliferating hepatocytes (ca. 40 hrs *versus* 20 hrs) (Fig. 6h, Supplementary Fig. 10a-b). This might indicate that those cells need a longer period to go through the S-phase checkpoints but eventually achieve mitosis. Thus, the concomitant inactivation of *PRKAR2A* enables *BAP1*-deficient hepatocytes to progress through S phase and divide, thereby allowing them to proliferate in a cAMP-stimulated ductal environment.

### Double *BAP1-* and *PRKAR2A*-mutant hepatocytes possess stemness and transdifferentiation capacity

**Fig. 7:**
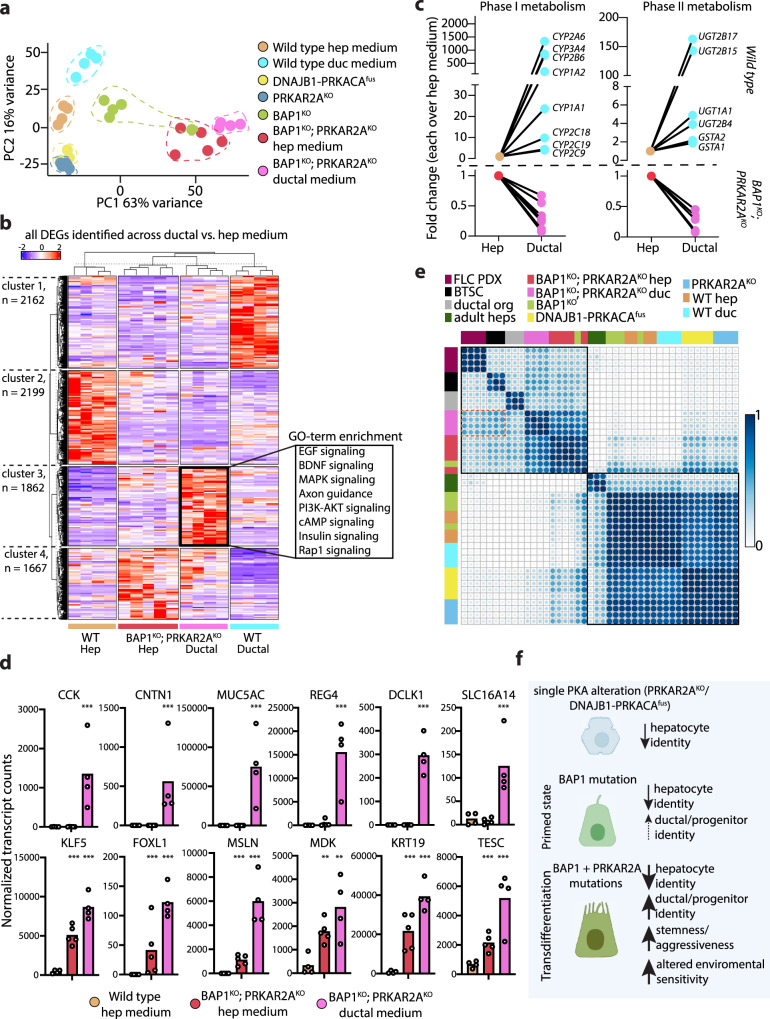
Concomitant loss of *BAP1* and *PRKAR2A* in hepatocytes induces cancer stemness. **a**, Principal component analysis based on whole transcriptome analysis of the indicated organoid lines cultured in hep or ductal medium. Each dot represents the transcriptome of a different clonal line. **b**, Heatmap depicting the hierarchical sample clustering and the expression of all differentially expressed genes across wild type organoids and *BAP1*^*KO*^*;PRKAR2A*^*KO*^ organoids cultured in hep medium *versus* ductal medium (|log2FC| > 0.5 and *P*-adj < 0.05, Wald test; DESeq2). GO-term enriched pathways are indicated for gene cluster 3 comparing *BAP1*^*KO*^*;PRKAR2A*^*KO*^ organoids in ductal *versus* hep medium. Each column is a different clonal line, in different media as indicated. **c**, Line plots of gene expression changes of differentially expressed genes (|log2FC| > 0.5 and *P*-adj < 0.05, Wald test; DESeq2) involved in phase I (left) and phase II (right) drug metabolism in wild type and *BAP1*^*KO*^*;PRKAR2A*^*KO*^ organoids cultured in ductal medium or hep medium. Data represent the mean fold change in ductal medium *versus* hep medium within its own line. n = 4 for wild type in both hep and ductal medium; *n* = 5 and 4 for *BAP1*^*KO*^*;PRKAR2A*^*KO*^ in hep and ductal medium, respectively. **d**, Bar plots of markers associated with stemness/progenitor identity and cancer-associated markers in *BAP1*^*KO*^*;PRKAR2A*^*KO*^ organoids cultured in ductal medium or hep medium as well as wild type organoids cultured in hep medium. Each dot indicates an independent organoid line and the mean of the normalized transcript counts from bulk RNA-sequencing is plotted. **P-adj < 0.01, ***P-adj < 0.001 *versus* wild type in hep medium (Wald test; DESeq2). n = 4, 5 and 4 for wild type, *BAP1*^*KO*^*;PRKAR2A*^*KO*^ in hep and ductal medium, respectively. **e**, Correlation plot of the transcriptomes of wild type organoids and the different mutant organoids in hep medium, and wild type and *BAP1*^*KO*^*;PRKAR2A*^*KO*^ organoid also in ductal medium, integrated with FLC PDX cultures, biliary tree stem cells (BTSC), adult human hepatocytes (all from Oikawa et al. ^21^) and EpCAM^+^ human liver ductal organoids (Artegiani et al. ^12^), using a gene set of the top 500 genes expressed each in the PDX FLC cultures, BTSCs, and adult hepatocytes (see Supplementary Data 3). **f**, Schematic summarizing the effect of FLC mutations on liver cell identity and associated features. Exact *P* values for **b** and **d** are provided in Supplementary Data 1. Source data are provided as a Source Data file.

We performed whole transcriptome analysis on wild type and *BAP1*^*KO*^;*PRKAR2A*^*KO*^ organoids cultured in ductal medium (*versus* hep medium) to understand the different molecular responses leading to cell cycle arrest in wild type cells, while this is overruled by the presence of the concomitant *BAP1* and *PRKAR2A* mutations. PCA on whole transcriptomes revealed a different direction upon culturing in ductal medium of wild type hepatocyte organoids as compared to *BAP1*^*KO*^;*PRKAR2A*^*KO*^ organoids, with the latter diverging even further away compared to the double mutants organoids cultured in hep medium (Fig. 7a). Clusters of differentially expressed genes in wild type and *BAP1*^*KO*^;*PRKAR2A*^*KO*^ organoids cultured in ductal *versus* hep medium were notably distinct (clusters 1 and 2 for wild type and clusters 3 and 4 for *BAP1*^*KO*^;*PRKAR2A*^*KO*^) (Fig. 7b, Supplementary Fig. 11a-b). In wild type hepatocytes, we noted that the ductal environment promoted the induction of many genes related to hepatocyte maturation, while genes related to proliferation and translation were repressed (Supplementary Fig. 11a,c,d). In particular, we found substantial upregulation of many metabolic enzymes involved in phase I and phase II drug metabolism, including cytochrome P450s (e.g. *CYP3A4, CYP2B6, CYP1A2*) and UDP-glycosyltransferases (UGTs), e.g. *UGT1A1, UGT2B17* (Fig. 7c), all expressed by mature hepatocytes. This response was inverted in the double mutant organoids. In fact, expression of most of these hepatocyte markers and functional genes were further repressed when switched from hep to ductal medium (Fig. 7c, Supplementary Fig. 11e). In vivo, PKA activation stimulates cholangiocyte proliferation^58^, while in hepatocytes it regulates lipid/sugar metabolism^59^. The proliferative nature of *BAP1*^*KO*^;*PRKAR2A*^*KO*^ organoids in a ductal (PKA-stimulated) environment suggests an acquired loss of sensitivity to hepatocyte differentiation cues and instead the adoption of a ductal/progenitor nature that enables sustained expansion.

We next focused on changes occurring in *BAP1*^*KO*^;*PRKAR2A*^*KO*^ organoids cultured in ductal medium. Amongst others, we found an enrichment for several pathways, including MAPK/EGF signaling (e.g. *EGFR, MAPK4, MAPK11*, *RPS6KA2*) and BDNF signaling (Fig. 7b, Supplementary Fig. 11f-g). Aberrant signaling of these pathways has been reported in FLC^11,34,36,43,60,61^. Interestingly, the double mutants also displayed deregulation of genes related to cAMP signaling as compared to wild type organoids and the other mutants, and particularly in response to the ductal medium (Supplementary Fig. 11g). This included induction of adenylate cyclase (*ADCY1*) and repression of PKA inhibitors, *PKIB* and *PKIG*, suggesting disruption of feedback loop mechanisms. The most notable changes of *BAP1*^*KO*^;*PRKAR2A*^*KO*^ cultured in ductal medium concerned the aggravation of the acquisition of cancer-associated features (Fig. 7d). These included specific induction of *CCK*, encoding the gastrointestinal, mitogenic peptide cholecystokinin, shown to elevate the growth of liver cancer cells^62^, as well as *CNTN1*, a neural cell-recognition molecule, whose expression was found as an unfavorable marker for liver cancer outcome^63^. Of note, BAP1-FLC tumors showed a selective enrichment of perineural invasion^11^. The enrichment for BDNF signaling and selective induction of signaling molecules such as *CNTN1* may facilitate this process. *MUC5AC* expression was also induced. Mucins are O-glycoproteins considered important players/drivers in liver cancer, especially in biliary tumors^64^. *DCLK1*, a tumor stem cell marker^65^, shown to be induced in liver cancer and associated with oncogenic reprogramming^66^, was also selectively induced in the double mutant cultured in ductal medium. Finally, we noted induction of *REG4*. In the pancreas, REG proteins facilitate acinar-to-ductal metaplasia^67^, and in this context REG4 could facilitate/enhance the observed hepatocyte-to-ductal transdifferentiation. Interestingly, some of the markers that have been reported for fusion-FLC tumors, were also induced in ductal medium (e.g. *TESC* and *SLC16A14*).

Notably, we detected expression of various stemness/progenitor markers as well as cancer-related markers in the double mutants already appearing in hep medium, but whose expression could often be further induced when switched to ductal conditions (Fig. 7d). These included, amongst others, *KRT19*, a ductal/progenitor marker, whose expression is associated with poor liver cancer prognosis^68^, as well as *FOXL1*, a marker of hepatic progenitor cells^69^. We further noted the selective and high induction of mesothelin (*MSLN*), a YAP target gene found enriched in a stemness cluster identified in a recent single-cell sequencing study of FLC-derived PDX^22^. We further noted increased expression of midkine (*MDK*), a growth factor associated with tumor progression^70^. Finally, we observed enhanced induction of *KLF5*, an important transcription factor determining stemness identity^71^. Altogether, the acquired loss of environmental sensitivity of *BAP1*^*KO*^;*PRKAR2A*^*KO*^ (a hallmark of cancer), could at least be partially explained by the adoption of a cancer stemness “ground” cell state.

To consolidate our observations of acquisition of liver ductal identity and stemness features, we compared the different organoids with 1) transcriptomes of human liver ductal organoids (derived from EpCAM^+^ cells)^12^ and 2) a transcriptomic dataset reporting on a long-term propagated PDX FLC model (that was reported to enrich for FLC cells with high stemness capacity)^21^, and additionally included transcriptomes of adult hepatocytes and biliary tree stem cells (BTSC) from the same study as comparison. Strikingly, only the double mutant *BAP1*^*KO*^;*PRKAR2A*^*KO*^ correlated highly with FLC PDX tissue and BTSC, and also to a lesser extent with human liver ductal organoids (Fig. 7e). This effect was more pronounced and selective when cultured in ductal medium as compared to in hep medium. Altogether, our findings suggest that the concomitant loss of *BAP1* and *PRKAR2A* drives human hepatocyte transdifferentiation into a ductal/progenitor like cancer stem cell that displays strong similarity to the cancer stem cell responsible for long-term propagation of FLC tissue in PDX models^21^ (Fig. 7f).

## Discussion

Understanding of the molecular pathogenesis of FLC remains obscure. Previous work has provided insights into the molecular characteristics of tumor tissues^5,6,10,11,17,36,37,43–45^, but mechanistic studies into how FLC mutations affect healthy liver cells and how different genetic backgrounds collectively drive FLC remains unknown. Relevant existing experimental human models include the growth of PDX lines and tumor organoids, both derived from a few primary or metastatic tumor samples^4,21–24^. Here, by using CRISPR-engineering in human hepatocyte organoids, we studied and compared the impact of introducing different FLC genetic backgrounds on the molecular and phenotypic behavior of human hepatocytes. This demonstrated the appearance of two molecular subgroups: one driven by a single PKA-related mutations (*i.e*. the *DNAJB1-PRKACA*^*fus*^ or single *PRKAR2A* loss) and the other driven by *BAP1* mutation.

How the chimeric DNAJ-PKAc fusion alters PKA signaling is not fully resolved. Multiple hypotheses have been proposed, including an altered substrate specificity and increased responsiveness to cAMP^4^. Effects of *PRKAR2A* loss in healthy liver are not well studied. We found that *DNAJB1-PRKACA*^*fus*^ and single *PRKAR2A*^*KO*^ induced remarkably similar changes in human hepatocytes. This may suggest that perturbations in the PKA holoenzyme could be similarly causative of FLC independently of the specific PKA mutation. This finding is also in line with the observation that FLC can develop in Carney complex patients harboring *PRKAR1A* mutations^7^.

The presence of *BAP1* mutations determined a distinct behavior of the targeted hepatocytes, and furthermore this mutation synergized with *PRKAR2A* inactivation to create a divergent cellular entity as compared to the single PKA-related mutants. By introducing simple genetic mutational landscapes (a single *DNAJB1-PRKACA* fusion or double *BAP1*^*KO*^*;PRKAR2A*^*KO*^ mutations) in a monocellular human hepatocyte system, the engineered organoids showed similarities and recapitulated some molecular features of the respective FLC tumors. Our comparative analysis revealed that all FLC mutations cause -to a certain extent- dedifferentiation of the hepatocytes. However, only the concomitant presence of both *BAP1* and *PRKAR2A* mutations leads to transdifferentiation into cells with liver ductal/progenitor identity. Similarly, both the fusion-FLC and the BAP1-FLC tumors display negative enrichment of the hepatocyte signature, while only the BAP1-FLC tumors positively enrich for the ductal/progenitor signature (Fig. 4b). Intriguingly, *BAP1*^*KO*^*;PRKAR2A*^*KO*^ organoids displayed a unique and completely different response when cultured in an environment promoting the growth of ductal cells. While wild type hepatocytes arrest their proliferation and thus further differentiate, double mutants respond to the same stimulus by boosting their proliferation and displaying enhanced cancer stemness properties. We previously showed that BAP1 loss has important consequences for the maintenance of epithelial characteristics in the context of CCA^12^. We hypothesize that the loss of BAP1 is instrumental to “prime” hepatocytes towards transdifferentiation, and that these primed cells, only upon concomitant genetic perturbation of PKA signaling, fully acquire an altered environmental sensitivity. This is also substantiated by our findings that single *BAP1*-mutant hepatocytes attempt to progress through the cell cycle in a ductal environment, but nevertheless encounter cell cycle arrest. PKA-related mutations are found among CCAs, including fusion events involving *PRKACA/B*^72,73^, while such mutations are rarely found in HCC, suggesting that PKA-related mutations can contribute to malignant ductal phenotypes. Importantly, cAMP-PKA signaling exhibits different effects in cholangiocytes and hepatocytes. While being a pro-proliferative factor in cholangiocytes^58^, in hepatocytes it instead controls mature functions including lipid and drug metabolism^59^. Single *PRKAR2A*^*KO*^ and *DNAJB1-PRKACA*^*fus*^ organoids mimicked wild type organoids by reducing their growth in response to cAMP stimulation, which could be explained by the fact that these cells harboring a single PKA mutation retained sufficient hepatocyte identity to respond in a hepatocyte-like fashion. *BAP1*^*KO*^ organoids instead did not alter their growth rate in response to cAMP stimulation, which would corroborate its cellular “primed” intermediate identity. Again, only the double mutant *BAP1*^*KO*^*;PRKAR2A*^*KO*^ responded with increased growth. We therefore hypothesize that FLC stemness might be linked to two prerequisites: a cell identity permissive to altered responses to signaling such as cAMP, and mutation-driven alterations in PKA signaling.

It was suggested that BAP1-driven FLC may have a cell-of-origin related to a hepato/pancreatic progenitor, based on transcriptomic changes and marker expression^11^. Our findings illustrate that the hepatocyte could instead equally well constitute the initially-targeted cell, given its transdifferentiation phenotype upon introduction of the *BAP1* and *PRKAR2A* mutations. In agreement, previous work has suggested that, under specific mutations, hepatocytes can constitute the tumor-initiating cell in CCA^74,75^. This implies that marker expression and transcriptomic correlation is thus not necessarily predictive to infer the cell-of-origin. The tumor-initiating cell is likewise debated for fusion-FLC tumors. Transcriptomic similarities suggested a progenitor cell as the originating cell, as well as the notion that the long-term propagation of PDX lines co-occurs with the appearance of a strong ductal/progenitor phenotype^17,21^. Instead, recreating the *DNAJB1-PRKACA* fusion using CRISPR-Cas9 in mouse hepatocytes via hydrodynamic tail vein injection resulted in the development of tumors in mouse liver, but with long latency (± 1 year) and lacking some typical FLC features, while co-mutating β-catenin (*CTNNB1*) accelerates tumor formation^19^. Engineering of *DNAJB1-PRKACA*^*fus*^ in human hepatocytes induced only some typical features also noted in FLC, such as a certain degree of hepatocyte dedifferentiation and a Wnt signature, but their cellular and molecular phenotypes were in general much milder with fewer cancer stemness features than what was observed in the *BAP1*^*KO*^*;PRKAR2A*^*KO*^ organoids. Fusion-driven and BAP1-driven FLC could be derived from a different cell-of-origin. However, another possible scenario is the need of additional mutation(s) to complement/synergize with the chimeric fusion for the appearance of a more cancer-like phenotype^76^, possibly through a similar hepatocyte transdifferentiation event as observed for *BAP1*^*KO*^*;PRKAR2A*^*KO*^. Indeed, various genomic studies have identified additional mutations in some fusion-driven FLC tumors, such as those in *TERT, CTNNB1*, or *MUC4*^6,77^. Finally, cell-to-cell contact with different liver cell types as well as changes in the niche environment or organ crosstalk may be important to ultimately drive cancer.

## Methods

### Human hepatocyte organoid culture

The use of human fetal liver for research was approved by the Dutch Ethical Medical Council (Leiden University Medical Center). Human hepatocyte organoids were established and cultured in Hep medium as described in detail previously^25,26,28^. Hep medium consists of AdvDMEM + ++ (AdDMEM/F-12 medium supplemented with 1× GlutaMAX, 10 mM HEPES and 100 U ml–1 penicillin/streptomycin solution (all Thermo Fisher)) supplemented with 15% RSPO1-conditioned medium (made in-house), 1x B-27 Supplement Minus Vitamin A (Gibco), 2.5 mM nicotinamide (Sigma), 1.25 mM N-acetyl-L-cysteine (Sigma), 50 ng/ml EGF (Peprotech), 50 ng/ml FGF7 (Peprotech), 50 ng/ml FGF10 (Peprotech), 50 ng/ml HGF (Peprotech), 20 ng/ml TGFα (Peprotech), 10 nM gastrin (Sigma), 3 μM CHIR-99021 (Sigma), 1 μM A 83-01 (Tocris), 5 μM Y-27632 (Sigma), and 50 μg/ml primocin (Invitrogen). Organoids were typically passaged every 7-10 days at a 1:2-1:4 ratio by manual pipetting with a P1000 pipette. During the first few days of organoid line establishment hep medium was supplemented with extra Y-27632 (final concentration: 10 μM) to minimize anoikis. Organoid lines were expanded every 7-10 days with a split ratio of 1:2-1:3. Brightfield images of all the lines were acquired regularly during culturing period.

### CRISPR-mediated gene knock-out and engineering of the *DNAJB1-PRKACA* chromosomal rearrangement

Organoids were CRISPR-engineered as described previously in Hendriks et al.^26^. NHEJ-mediated knock-out was exploited to generate *BAP1* and *PRKAR2A* knock-out mutants, while a sgRNA-mediated double-cut strategy was used to eliminate a 400 kb region and generate an endogenous fusion between *DNAJB1* and *PRKACA* genes. Single-guide (sg)RNAs were designed using an online web-tool (www.atum.bio/eCommerce/cas9/input) and cloned into the pSPgRNA plasmid (Addgene #47108) as previously described by Ran et al. ^78^. The sgRNA sequences used in this study are given in Supplementary Data 4. The appropriate sgRNA plasmids were co-transfected with a plasmid expressing SpCas9 as well as mCherry for visualization of transfected cells (Addgene #66940). To select for transfected cells, we employed an in-house two-plasmid transposon system comprised of a *piggyBac* transposase and a donor plasmid with terminal repeats bearing a cassette with a hygromycin resistance gene. Human hepatocyte organoids were transfected by electroporation as described in detail elsewhere^26^.When organoids were co-transfected with the hygromycin resistance gene-*piggyBac* two plasmid system, selection (Hygromycin B Gold (100 μg/ml, InvivoGen)) was started when organoids of small size had formed after electroporation (typically after 7 days) and hygromycin was kept in the culture medium until selection was complete (ca. 7-12 days). Single surviving organoids were picked, made into small fragments/single cells by Accutase, plated into a single BME droplet well of a 24-well plate, expanded into clonal lines and genotyped to confirm the presence of the desired mutation. Multiple clonal lines (at least 3) for each mutant were generated and used for analysis. For the generation of the FUCCI-reporter lines, we electroporated the different mutant lines using a two-plasmid transposon system comprised of a *piggyBac* transposase and a donor plasmid with terminal repeats bearing a cassette with the FUCCI construct (Clover-Geminin-IRES-mKO2-Cdt1, Addgene #133371). After electroporation, outgrowing organoids displaying fluorescence were manually picked, made into small fragments/single cells by Accutase, plated into a single BME droplet well of a 24-well plate, and expanded into FUCCI lines that were used for downstream analysis.

### Genotyping of mutant lines

DNA was extracted from the clonal organoid lines using a lysis buffer (0.1 M Tris-HCl pH 8.5, 0.2 M NaCl, 0.2% SDS, 0.05 M EDTA, 0.4 mg/ml proteinase K) and isopropanol precipitation as described^26^. To genotype the *BAP1* and/or *PRKAR2A* knock-out mutants, PCR reactions were performed to amplify the genomic region encompassing the sgRNA/Cas9-targeted area. PCR products were Sanger sequenced to confirm the genotypes. Genotypes were deconvoluted using the ICE v2 CRISPR tool and PCR products were further subcloned to discriminate between alleles, whenever appropriate. Lines carrying frameshift indels were selected and further used for culturing. To genotype the *DNAJB1-PRKACA* fusion, we used primers spanning the 400 kb deletion sequence and screened the lines for the presence of PCR amplicons (see the Source data file for the uncropped gel and the expected amplicon size, related to Fig. 1d). Positive ones were PCR-purified and Sanger sequenced to confirm the presence of the fusion event. Genotyping primers are listed in Supplementary Data 4.

### Histology, whole-mount immunofluorescence, confocal imaging, and quantification

For H&E staining, organoids were washed from the BME using 1X PBS, fixed overnight in 4% PFA, and embedded in paraffin blocks. 5 μm sections were processed for H&E staining and imaged with a Leica DM6 microscope. For immunofluorescence analysis, organoids were washed from the BME using cold 1X PBS and then fixed by incubation with 4% formaldehyde for 2 hrs. Samples were then washed 2 times with 1X PBS and kept at 4 °C before further processing. For each staining, we used organoids obtained from a minimum of 1 well of a 24-well plate. Stainings were performed on whole organoids contained in a 1.5 ml tube (Eppendorf) by an initial permeabilization and blocking incubation step of 2 hrs at room temperature in blocking buffer (1X PBS, 5% BSA, 0.02% Triton-X-100), and an overnight primary antibody incubation at 4 °C with the appropriate primary antibody at the indicated dilutions (Anti-Ki67 (SolA15), Thermo Fisher, #14-5698-82, dilution 1:1000; Phalloidin-Atto 647 N, Sigma, #65906, dilution 1:1000; Anti-beta catenin, Santa Cruz, #sc-7199, dilution 1:1000; Anti-ZO1, Thermo Fisher, #PA5-19090, dilution 1:500; Anti-ALB, Thermo Fisher, #A80-229A, dilution 1:300; Anti-pan-cytokeratin, BD Biosciences, #345779, dilution 1:500; Anti-cleaved caspase-3 (Asp175), Cell Signaling Technology, #9661 L, dilution 1:400; Anti-pCREB (Ser133) (87G3), Cell Signaling Technology, #9198 S, dilution 1:400; Anti-EMA, Abcam, #ab15481, dilution 1:400; Anti-CD44 (G44-26), BD Biosciences, #555476, dilution 1:400; Anti-KRT7 (OV-TL 12/30), Thermo Fisher, #MA5-11986, dilution 1:400; Anti-KRT19 (D7F7W), Cell Signaling Technology, #13092 S, dilution 1:500) in incubation buffer (2% BSA-1X PBS). Organoids were washed 3 times with 1X PBS and incubated for 4 hrs at 4 °C with appropriate secondary antibodies diluted 1:1000 in incubation buffer. Organoids were then incubated with DAPI (1 μg/ml) diluted in 1X PBS for 20 min at room temperature to counterstain the nuclei. Organoids were washed 3 times with 1X PBS and transferred to a 96-well glass-bottom plate (SensoPlate) and mounted using Immu-Mount mounting medium (Thermo Fisher). For staining of intracellular lipids, organoids were stained with Nile Red (0.5 μg/ml, Thermo Fisher) and DAPI (1 μg/ml, Thermo Fisher) for 20 min. Organoids were then washed 2 times with 1X PBS and transferred to a 96-well glass-bottom plate. All organoids were imaged with a 10X or 20X objective on a Sp8 confocal microscope (Leica). Lif files were processed using Leica LAS X software (v1.1) and analyzed for measurement of mean fluorescence intensity, ROI areas and manual counting of positive cells for any given markers, using ImageJ (v1.53) and Photoshop CS4 (v24.1.1). Images obtained from at least 2 different clonal lines for each mutation were acquired and quantified for each staining.

### Growth assessment in a ductal cell/progenitor environment

To assess the growth of wild type as well as the different mutant organoid lines in an environment stimulating the growth of ductal/progenitor cells, we switched the medium from hep medium to ductal medium^48^, which consisted of AdDMEM + ++ supplemented with 1x B-27 Supplement Minus Vitamin A, 10 mM nicotinamide, 1.25 mM N-acetyl-L-cysteine, 10 nM gastrin, 50 ng/ml EGF, 10% RSPO1-conditioned medium, 100 ng/ml FGF10, 25 ng/ml HGF, 5 μM A83-01, 10 μM forskolin (Tocris), and 50 μg/ml primocin. Organoids were dissociated into single cells by Accutase digestion, plated into a single BME droplet well of a 24-well or 96-well plate and cultured in hep or ductal medium. We characterized the acute growth characteristics of all lines by ATP-based viability assay (CellTiter-Glo 3D, Promega) over a time-course of 14 days. Brightfield images of all the lines were acquired regularly during culturing period. Organoid line maintenance in ductal medium was followed over a period of 50 days. Only the *BAP1*^*KO*^*;PRKAR2A*^*KO*^ double mutant could grow in ductal medium and were therefore expanded every 7-10 days with a split ratio of 1:3-1:4.

### PKA stimulation experiments

To characterize the baseline PKA activity of the different organoid lines and in response to a pulse of stimulation (72 h exposure to the adenylate cyclase activator forskolin (10 μM)) we evaluated pCREB levels. To this end, organoids (baseline and forskolin-stimulated) were processed for immunofluorescence as described below. pCREB levels were assessed via two means: 1) the pCREB fluorescence intensity across each organoid and 2) the amount of pCREB+ cells within the same organoid. Both measurements were either used to plot these values for a matching organoid on a *x*-*y* axis, or alternatively these values were multiplied to constitute the “pCREB level”. To assess the growth capacity of the different organoid lines under chronic PKA stimulation, organoids were dissociated into single cells by Accutase digestion, plated into a single BME droplet well of a 24-well plate and cultured in hep medium or hep medium supplemented with forskolin (10 μM). Outgrowing organoids were split at the same ratio over a period of 50 days. Brightfield pictures were acquired at regular intervals and after 50 days organoids were collected for phenotypic characterization.

### Live imaging and FUCCI-based cell cycle measurements

For time-lapse microscopy imaging, FUCCI-transfected organoid lines were passaged 4 days before imaging and seeded onto glass-bottom plates (Greiner, #662892) and cultured in hep or ductal medium, as indicated. All imaging was performed using an Sp8 confocal microscope (Leica), which was continuously held at 37 °C and equipped with a culture chamber for overflow of 5% CO_2_. Imaging of the wild type, *BAP1*^*KO*^, and *BAP1*^*KO*^*;PRKAR2A*^*KO*^ double mutant hepatocyte organoids was performed for up to 72 h, with acquisition intervals of 30 min and z-stack steps of 4 μm. For fluorescence detection, we used minimal amounts of excitation light. ImageJ was used to assemble the videos and for image analysis. At least 10 organoids for each condition were imaged and quantified. For fluorescence intensity analysis, individual cells were defined as ROI and the ROI was adjusted manually over time due to cell movement. Fluorescence in the 488 and 568 channels was measured for each ROI and ROI were defined for each time interval. The fluorescence intensity for the two channels was normalized internally for each individual cell based on the highest fluorescence intensity. Results derived from multiple cells displaying the same cell cycle dynamics were averaged and plotted using GraphPad Prism (v8.2.0).

### RNA extraction and qPCR

To isolate RNA, 1 well of a 12-well plate containing organoids was washed twice with cold 1X PBS to remove BME, and collected in 1 ml TRIzol Reagent (Thermo Fisher). Samples were fully lysed by repetitive pipetting with a P1000 pipette and then snap-frozen in liquid nitrogen. RNA was extracted using isopropanol precipitation using the TRIzol manufacturer’s protocol and resuspended in sterile water. Extracted RNA was stored at −80 °C. For qPCR analysis, RNA concentrations and purity were determined using a NanoDrop spectrophotometer. Then, 250 ng of RNA was used for cDNA production using the SuperScript IV kit (Thermo Fisher). The cDNA reaction was diluted 1:10, and 2 μl of the diluted cDNA was used for each qPCR reaction. qPCR reactions were performed using the iQSYBRGreen mix (Bio-rad). To detect the expression of the *DNAJB1-PRKACA* fusion, we used a qPCR fw primer in exon 1 of *DNAJB1* and a qPCR rev primer spanning the exon 2-exon 3 junction of *PRKACA*). qPCR primers are listed in Supplementary Data 4. For each experiment and each organoid line, technical triplicates were performed.

### Bulk RNA-sequencing and bioinformatic analysis

For bulk RNA-sequencing, RNA was extracted from wild type and mutant organoids cultured in hep medium. To probe the effect of ductal medium, RNA from wild type and *BAP1*^*KO*^*;PRKAR2A*^*KO*^ organoids was isolated after 1 month in ductal medium. RNA integrity was measured using the Agilent RNA 6000 Nano kit with the Agilent 2100 Bioanalyzer and RNA concentrations were determined using the Qubit RNA HS Assay Kit. RIN values of RNA samples were typically 9.5–10 and only samples with RIN > 9 were used for libraries preparation. RNA libraries were prepared using TruSeq Stranded mRNA polyA kit (Illumina) and paired-end (2×50 bp) sequenced on an Illumina Nextseq 2000. Library preparation and sequencing was performed by USEQ (Utrecht Sequencing Facility). Quality control (QC) on the sequence reads from the raw FASTQ files was done with FastQC. TrimGalore was used to trim reads based on quality and adapter presence after which FastQC was again used to check the resulting quality. rRNA reads were filtered out using SortMeRNA after which the resulting reads were aligned to the reference genome fasta (genome.fa) using the STAR aligner. Follow-up QC on the mapped (bam) files was done using Sambamba, RSeQC and PreSeq. Read counts were then generated using the Subread FeatureCounts module with the Homo_sapiens.GRCh37.74.gtf file as annotation. From the dataset, lowly expressed genes were filtered out (<100 transcript counts across all samples) and transcript normalization was performed using the DESeq2 package^79^ in RStudio (v2022.02.2). Differential gene expression analysis was performed with DESeq2 (v3.16) and considered log2 fold changes and statistical significance are indicated throughout the manuscript. Calculation and visualization of principal components for bulk RNA-sequencing was computed using the R package factoextra (v1.0.7). Unsupervised hierarchical clustering was performed to identify groups of genes having similar expression profiles across samples and to classify mutants based on gene expression similarities. Based on whole transcriptome clustering, one *DNAJB1-PRKACA*^*fus*^ sample clearly clustering apart from all other fusion organoids was defined as an outlier and excluded from further analysis. Heatmaps presented in this study were generated using the ComplexHeatmap package (v3.16). Gene Ontology enrichment analyses were performed using Enrichr (https://maayanlab.cloud/Enrichr/). Gene set enrichment analysis (GSEA, v4.2.3) to define cell identity was performed using gene sets for ductal cell/progenitor cell signatures and hepatocyte signatures, as reported in Aizarani et al.^49^ (Supplementary Data 3). Normalized transcript counts or fold changes *versus* wild type organoids were used for analysis and data visualization. Data visualization was performed either using ggplot2 (v3.4.1) in RStudio or GraphPad Prism.

### Comparison of mutant organoids with primary FLC tumor tissues

To address similarities between the engineered FLC-mutant organoids and primary FLC tumor tissues, we made comparisons with the transcriptomic datasets on fusion-FLC tumors, BAP1-FLC tumors, and normal, healthy livers from Hirsch et al.^11^. We merged the organoid dataset with the tumor dataset and performed batch correction by removing genes detected/expressed only in one of the two datasets. We then filtered out lowly expressed genes (<100 transcript counts across all samples) and performed transcript normalization as described above. Correlations were calculated using the Corrplot package and dendograms were generated using the dendextend (v1.16.0) in RStudio. Gene set enrichment analysis to assess organoid-tumor similarity was performed using gene sets of both upregulated and downregulated genes (|log2FC| > 0.5 and *P*-adj. < 0.01 (Wald test; DESeq2)) observed in fusion-FLC tumors or BAP1-FLC tumors, to compare the *DNAJB1-PRKACA*^*fus*^ organoids and *BAP1*^*KO*^*;PRKAR2A*^*KO*^ organoids, respectively. Additionally, we used detected genes from a fusion-FLC tumor signature defined by Lalazar et al.^23^ to evaluate mutant organoid-tumor similarities.

### Reporting summary

Further information on research design is available in the Nature Portfolio Reporting Summary linked to this article.

## Supplementary information

Supplementary Information

Description of Additional Supplementary Files

Supplementary Data 1

Supplementary Data 2

Supplementary Data 3

Supplementary Data 4

Reporting Summary

## Data Availability

Bulk RNA-sequencing data generated in this study have been deposited in the GEO database under accession code GSE215785. All other data are available in the main text, its Supplementary information files or in the source data file, or from the corresponding authors upon reasonable request. Source data are provided with this paper.

## Source data

Source Data
