# Supplementary material for: Organoid models of fibrolamellar carcinoma mutations reveal hepatocyte transdifferentiation through cooperative BAP1 and PRKAR2A loss
Source: Nat Commun. 2023 May 3;14:2377. doi: 10.1038/s41467-023-37951-6 (PMC10156813; doi:10.1038/s41467-023-37951-6)
Supplement: Supplementary file 1 — Supplementary Information [file 41467_2023_37951_MOESM1_ESM.pdf]

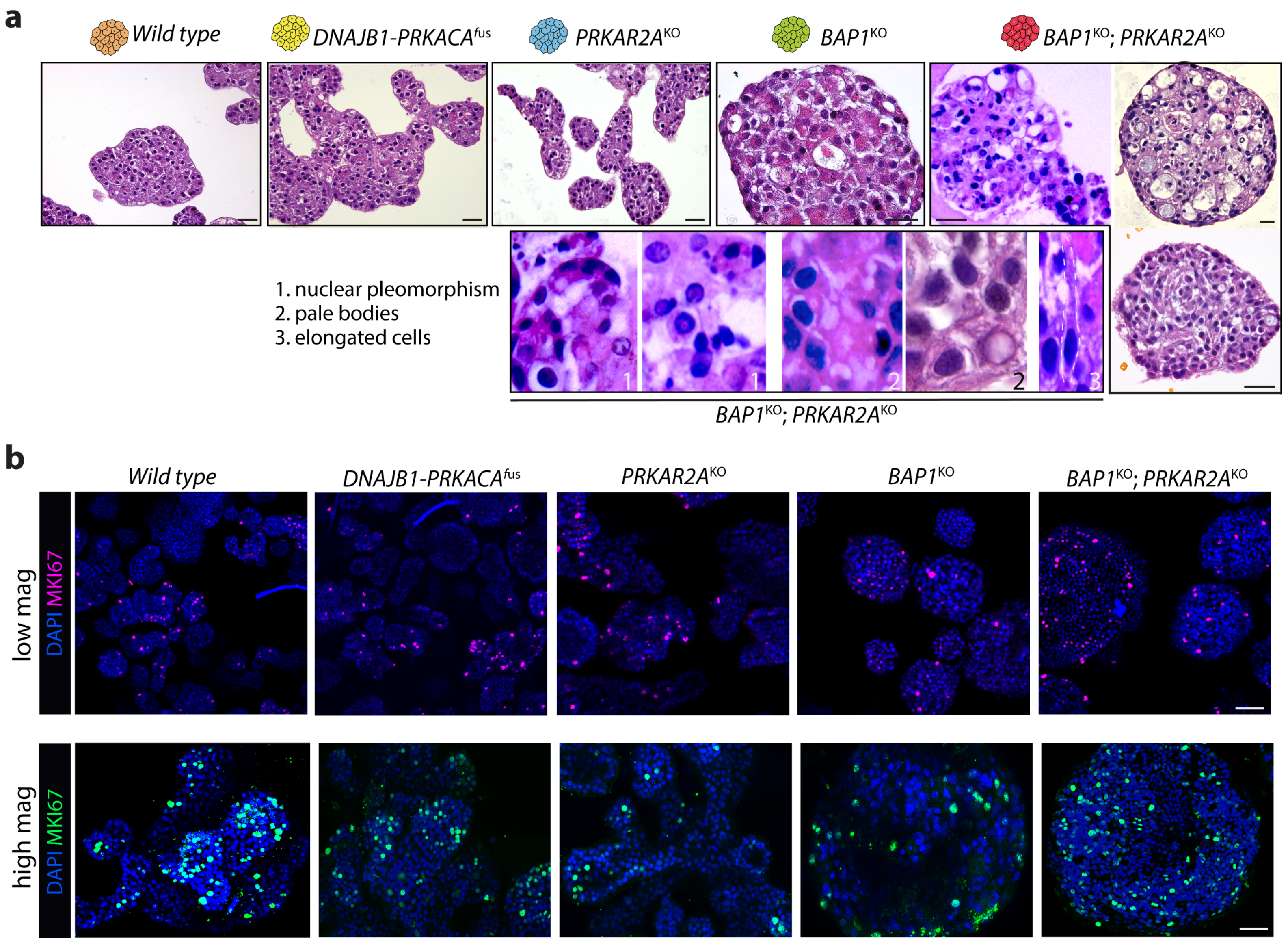

Supplementary Figure 1. Histological characterization of human hepatocyte organoids engineered to carry FLC mutations.

a, Representative images of H&E staining of the different FLC mutant organoid lines. Examples of relevant cellular features for the double mutant are highlighted in the inset. Scale bar = 20 μm.

b, Representative images of MKI67 staining at lower (top) and higher (bottom) magnification of the different FLC mutant organoid lines. Scale bar = 100 μm (low magnification) and 50 μm (high magnification).

a-b, Both representative of 3 independent lines per genotype.

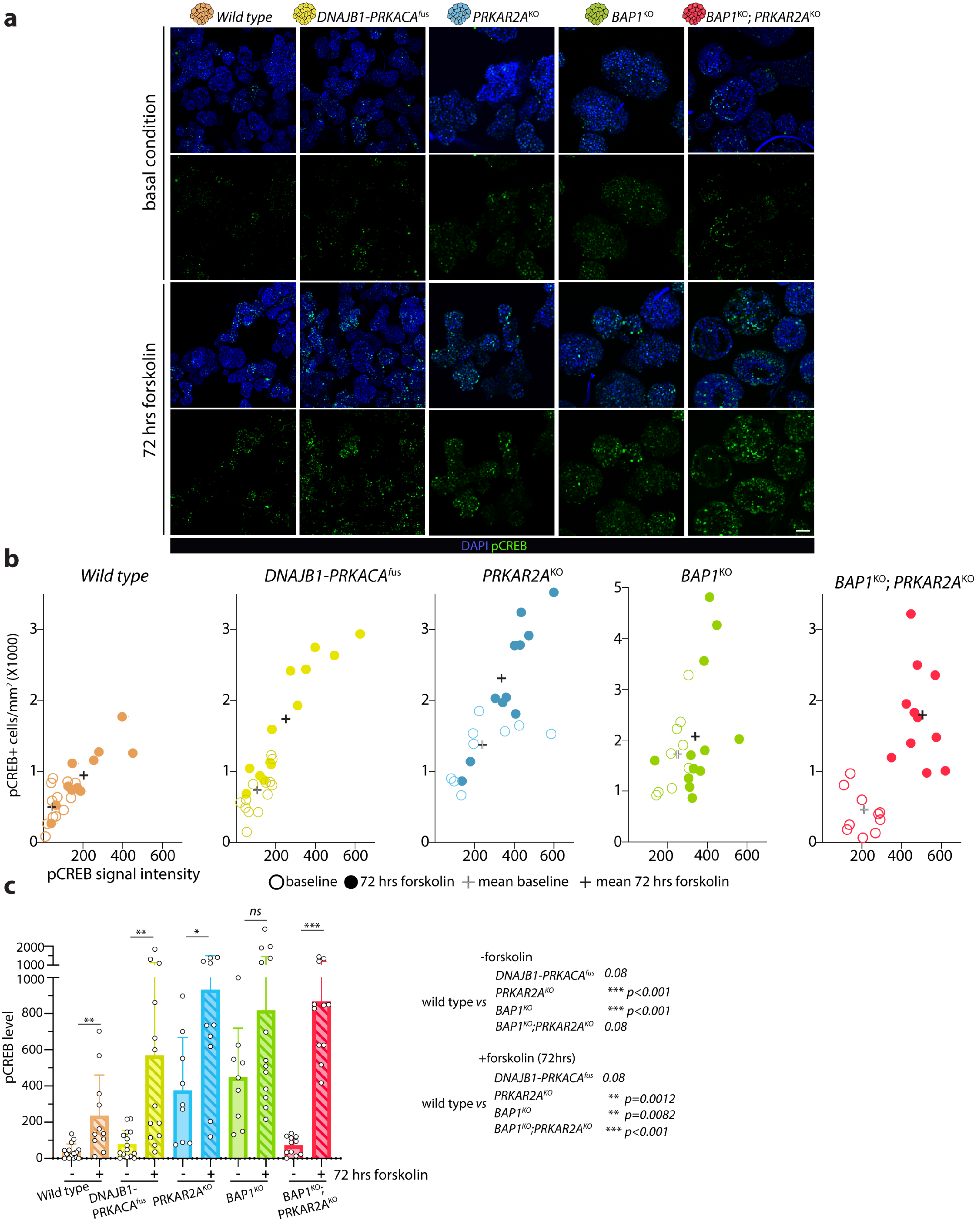

a
Wild type
DNAJB1-PRKACA^fus
PRKAR2A^KO
BAP1^KO
BAP1^KO; PRKAR2A^KO
basal condition
72 hrs forskolin
DAPI pCREB
b
pCREB+ cells/mm² (X1000)
pCREB signal intensity
baseline
72 hrs forskolin
mean baseline
mean 72 hrs forskolin
c
pCREB level
72 hrs forskolin
Wild type
DNAJB1-PRKACA^fus
PRKAR2A^KO
BAP1^KO
BAP1^KO; PRKAR2A^KO
ns
-forskolin
wild type vs
DNAJB1-PRKACA^fus 0.08
PRKAR2A^KO *** p<0.001
BAP1^KO *** p<0.001
BAP1^KO;PRKAR2A^KO 0.08
+forskolin (72hrs)
wild type vs
DNAJB1-PRKACA^fus 0.08
PRKAR2A^KO ** p=0.0012
BAP1^KO ** p=0.0082
BAP1^KO;PRKAR2A^KO *** p<0.001

Supplementary Figure 2. Characterization of PKA activity across the FLC-mutant and wild type organoid lines.
a, Representative images of pCREB (Ser133) staining of organoids in homeostasis (top) and after 72 h exposure (bottom) to the cAMP activator forskolin (FSK) for the different FLC mutant organoid lines. Scale bar = 50 μm.
b, Scatter plot showing quantification of pCREB level across the mutants in baseline (open circles) and upon forskolin stimulation (closed circles). Number of pCREB+ cells (over organoid area) is shown on the y-axis and the pCREB signal intensity over the whole organoid is shown on the x-axis. Each dot represents an organoid (in which both measurements were performed). 3 independent lines per genotype were quantified. Mean is shown with the indicated symbols.
c, Bar plot showing the pCREB level (defined as number of pCREB+ cells multiplied by the pCREB signal intensity) across mutants, as in **b**. Mean ± SD is plotted. Statistical significance between unstimulated versus stimulated is shown in the plot, while statistical significance between the different conditions versus wild type is provided to the right. Sample size as in b. $^{*}P < 0.05$, $^{**}P < 0.01$, $^{***}P < 0.001$; two-tailed Student's t-test. Exact P values are provided in the source data.

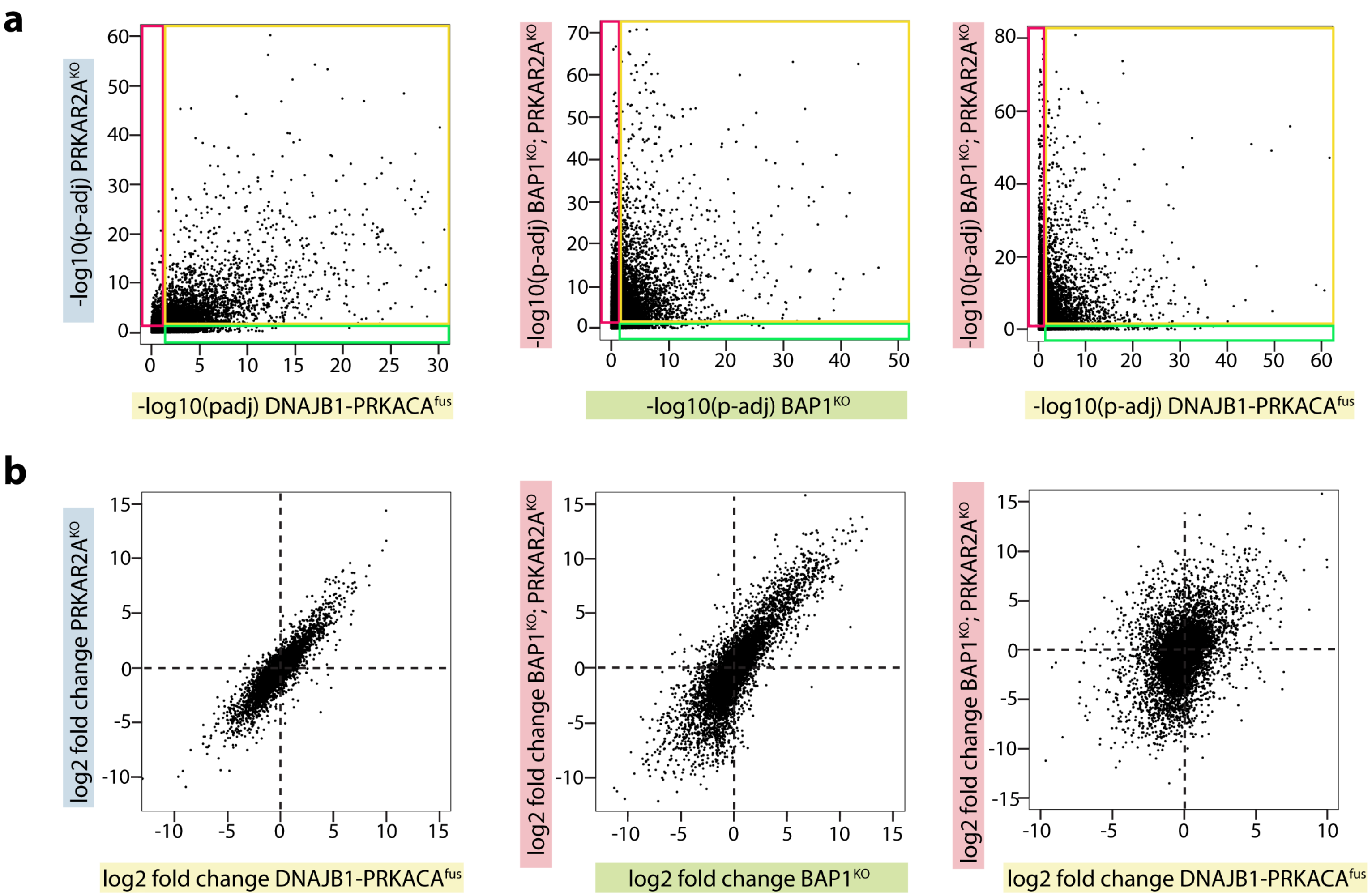

Supplementary Figure 3. Transcriptomic commonalities and differences between FLC mutant organoid models.

a, Dot plots showing the common (yellow box) and exclusive (red and green boxes) differentially expressed genes based on P-adj values (Wald test; DESeq2) within PRKAR2A^{KO} and DNAJB1-PRKACA^{fus} (left), BAP1^{KO};PRKAR2A^{KO} and BAP1^{KO} (middle) and BAP1^{KO};PRKAR2A^{KO} and DNAJB1-PRKACA^{fus} (right). Each dot represents a gene.

b, Correlation plots showing log2FC correlations between PRKAR2A^{KO} and DNAJB1-PRKACA^{fus} (left), BAP1^{KO};PRKAR2A^{KO} and BAP1^{KO} (middle) and BAP1^{KO};PRKAR2A^{KO} and DNAJB1-PRKACA^{fus} (right). Each dot represents a gene.

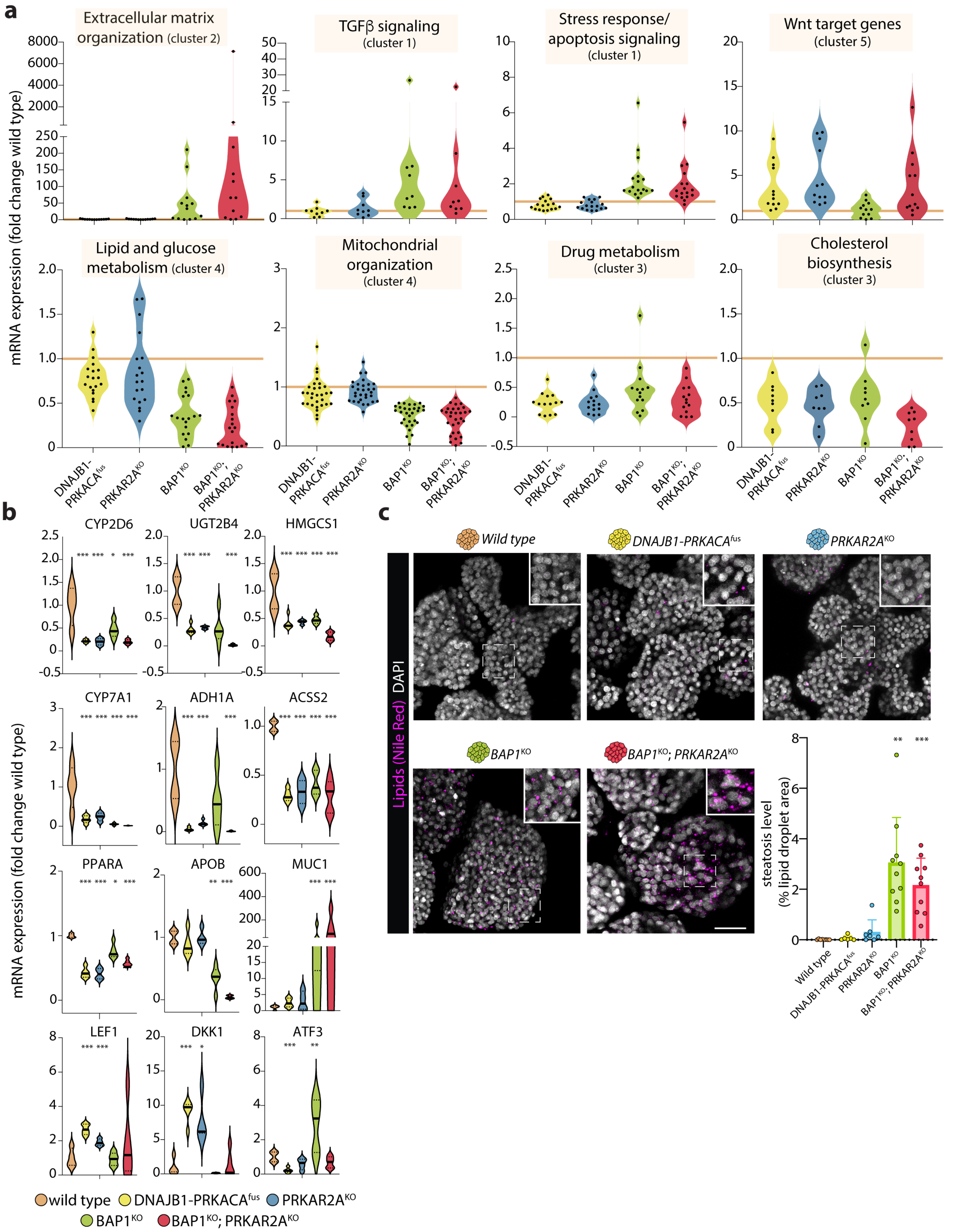

a
Extracellular matrix organization (cluster 2)
TGFβ signaling (cluster 1)
Stress response/ apoptosis signaling (cluster 1)
Wnt target genes (cluster 5)
Lipid and glucose metabolism (cluster 4)
Mitochondrial organization (cluster 4)
Drug metabolism (cluster 3)
Cholesterol biosynthesis (cluster 3)
mRNA expression (fold change wild type)
DNAJB1-PRKACA^fus
PRKAR2A^KO
BAP1^KO
BAP1^KO; PRKAR2A^KO
b
CYP2D6
UGT2B4
HMGCS1
CYP7A1
ADH1A
ACSS2
PPARA
APOB
MUC1
LEF1
DKK1
ATF3
mRNA expression (fold change wild type)
wild type
DNAJB1-PRKACA^fus
PRKAR2A^KO
BAP1^KO
BAP1^KO; PRKAR2A^KO
c
Wild type
DNAJB1-PRKACA^fus
PRKAR2A^KO
BAP1^KO
BAP1^KO; PRKAR2A^KO
Lipids (Nile Red) DAPI
steatosis level (% lipid droplet area)
Wild type
DNAJB1-PRKACA^fus
PRKAR2A^KO
BAP1^KO
BAP1^KO; PRKAR2A^KO

Supplementary Figure 4. Transcriptomic and functional alterations in FLC mutant organoids.

a, Violin plots showing the fold change expression of specific genes belonging to the indicated pathways across the different FLC mutant organoids relative to the expression in wild type organoids. Each dot represents a different gene. For the list and mean fold changes of genes plotted per group, see **Supplementary Data 2**.

b, Violin plots showing the expression of selected genes across the different FLC mutant organoids relative to the expression in wild type organoids. *P-adj < 0.05, **P-adj < 0.01, ***P-adj < 0.001 versus wild type (Wald test; DESeq2). Exact P values are provided in **Supplementary Data 1**.

c, Representative images of intracellular lipid accumulation using Nile Red staining in the different FLC mutant organoids. The bar plot shows the lipid droplet area coverage within an organoid (DAPI+). Mean ± SD is plotted. 3 independent lines per genotype were quantified. ***P < 0.001 versus wild type; two-tailed Student's t-test. Exact P values are provided in the source data. Scale bar = 50 μm.

Source data are provided as a Source Data file.

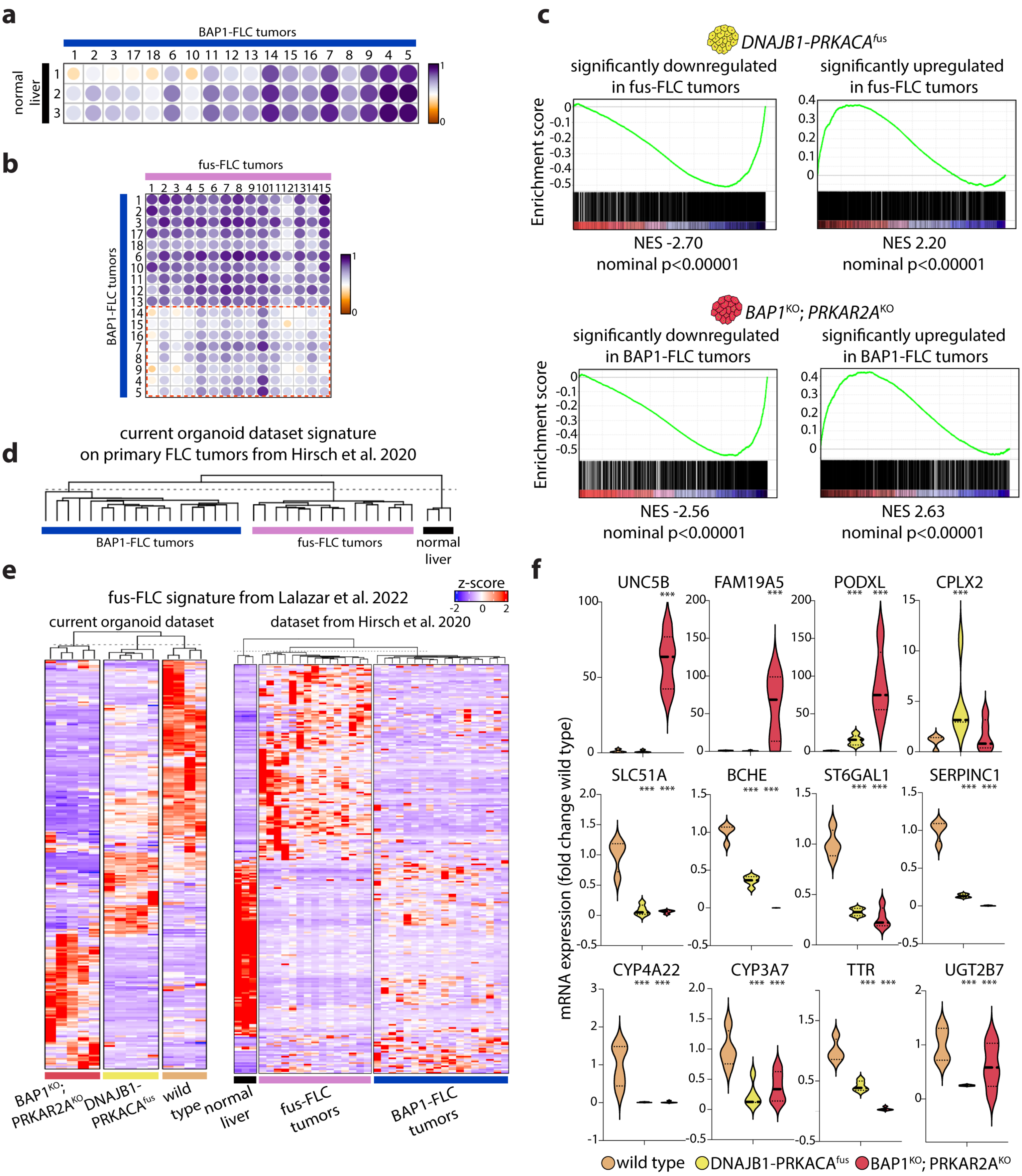
a
BAP1-FLC tumors
normal liver
b
fus-FLC tumors
BAP1-FLC tumors
c
DNAJB1-PRKACA^fus
significantly downregulated in fus-FLC tumors
significantly upregulated in fus-FLC tumors
Enrichment score
NES -2.70
nominal p<0.00001
NES 2.20
nominal p<0.00001
BAP1^KO; PRKAR2A^KO
significantly downregulated in BAP1-FLC tumors
significantly upregulated in BAP1-FLC tumors
NES -2.56
nominal p<0.00001
NES 2.63
nominal p<0.00001
d
current organoid dataset signature on primary FLC tumors from Hirsch et al. 2020
BAP1-FLC tumors
fus-FLC tumors
normal liver
e
fus-FLC signature from Lalazar et al. 2022
z-score
current organoid dataset
dataset from Hirsch et al. 2020
BAP1^KO; PRKAR2A^KO
DNAJB1-PRKACA^fus
wild type
normal liver
fus-FLC tumors
BAP1-FLC tumors
f
UNC5B
FAM19A5
PODXL
CPLX2
SLC51A
BCHE
ST6GAL1
SERPINC1
CYP4A22
CYP3A7
TTR
UGT2B7
mRNA expression (fold change wild type)
wild type
DNAJB1-PRKACA^fus
BAP1^KO; PRKAR2A^KO

Supplementary Figure 5. Engineered FLC-mutant organoids share similarities with FLC tumor tissues.
a, Correlation plot using whole transcriptomes comparing healthy normal liver with BAP1-FLC tumors (re-analyzed from Hirsch *et al*.).
b, Correlation plot using whole transcriptomes comparing BAP1-FLC tumors with fusion-FLC tumors (re-analyzed from Hirsch *et al*.).
c, GSEA using all upregulated or downregulated genes (|log2FC| > 0.5 and P-adj < 0.01, Wald test; DESeq2) identified in fusion-FLC tumors or BAP1-FLC tumors (versus normal liver, see Supplementary Table 3) on DNAJB1-PRKACAfus organoids and BAP1^{KO};PRKAR2A^{KO} organoids, respectively, both versus wild type. Normalized enrichment scores (NES) and nominal P values are indicated in the figure panels.
d, Unsupervised hierarchical clustering of normal liver, BAP1-FLC tumors, and fusion-FLC tumors using a signature based on the current organoid mutant dataset (all differentially expressed genes identified across all mutants versus wild type), demonstrating distinct clustering of healthy liver versus each respective FLC tumor subtype. Each line of the tree represents a sample.
e, Unsupervised hierarchical clustering of the FLC mutant organoids using the fusion-FLC signature previously described by Lalazar *et al*.. The same signature was also used to cluster the transcriptomes of normal liver and the two FLC tumor subtypes from Hirsch *et al*.. Note identical clustering for the type of organoid mutants as well as for the type of primary samples. Each column represents a sample.
f, Violin plots showing the expression of reported downregulated FLC markers in DNAJB1-PRKACA^{fus} and BAP1^{KO};PRKAR2A^{KO} organoids relative to the expression in wild type organoids. ***P-adj < 0.001 (Wald test; DESeq2). Exact P values are provided in **Supplementary Data 1**.
Source data are provided as a Source Data file.

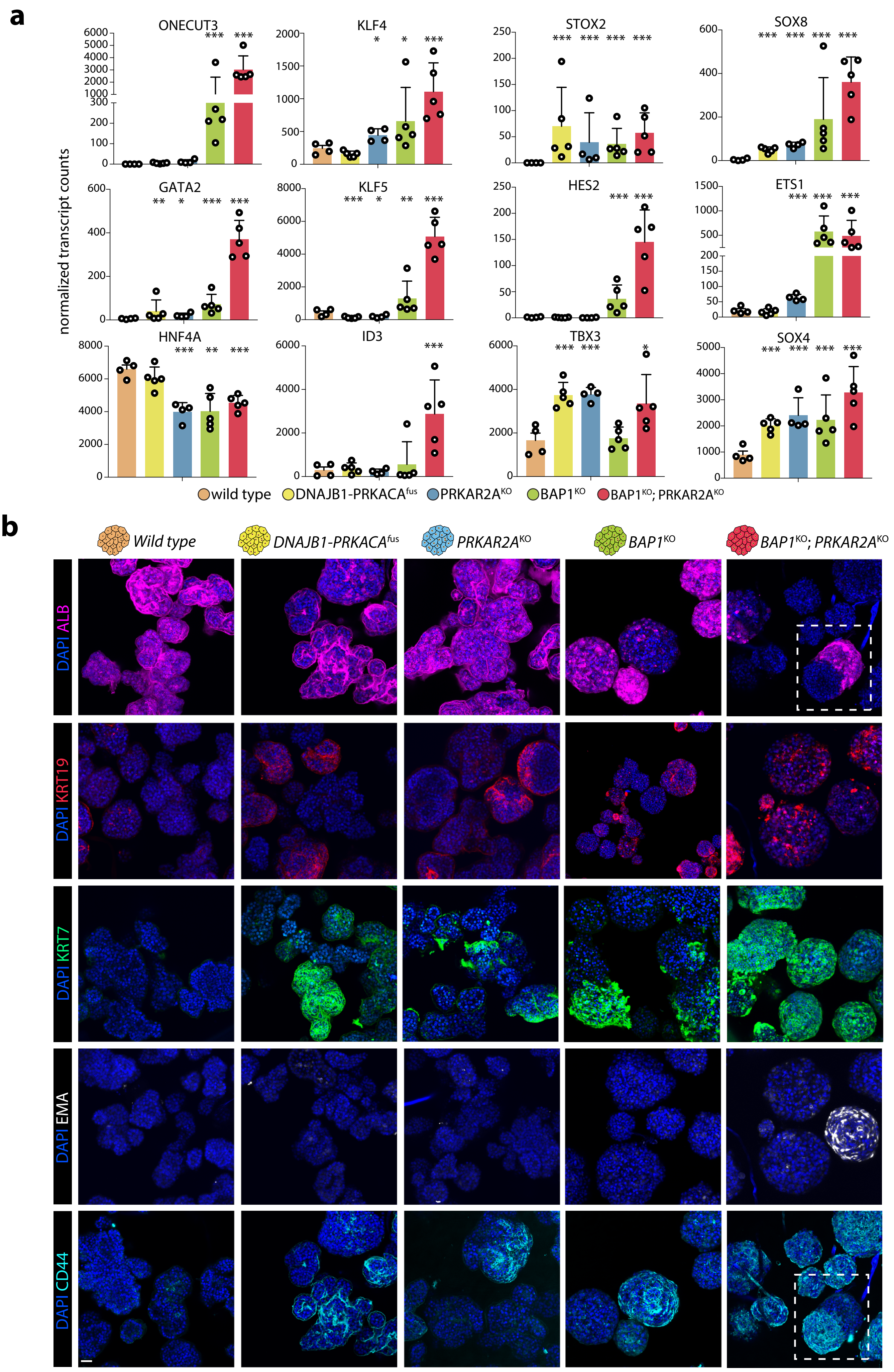
a
ONECUT3
KLF4
STOX2
SOX8
GATA2
KLF5
HES2
ETS1
HNF4A
ID3
TBX3
SOX4
normalized transcript counts
wild type
DNAJB1-PRKACA^fus
PRKAR2A^KO
BAP1^KO
BAP1^KO; PRKAR2A^KO
b
Wild type
DNAJB1-PRKACA^fus
PRKAR2A^KO
BAP1^KO
BAP1^KO; PRKAR2A^KO
DAPI ALB
DAPI KRT19
DAPI KRT7
DAPI EMA
DAPI CD44

Supplementary Figure 6. Transcriptional program alterations and phenotypic changes in the FLC mutant organoid models.

a, Bar plots demonstrating the expression of different transcriptional factors in the different FLC mutant organoid models in comparison to the expression in wild type organoids. Normalized transcript counts derived from bulk RNA-sequencing are plotted. Each dot represents expression in an independent organoid line. Mean ± SD is plotted. *P-adj < 0.05, **P-adj < 0.01, ***P-adj < 0.001 versus wild type (Wald test; DESeq2). n = 4, 5, 4, 5 and 5 for wild type, DNAJB1-PRKACA^{fus}, PRKAR2A^{KO}, BAP1^{KO} and BAP1^{KO};PRKAR2A^{KO}, respectively. Exact P values are provided in **Supplementary Data 1**.

b, Representative high magnification images of staining for hepatocyte (ALB), ductal/biliary (KRT7/19), and tumor (CD44 and EMA) markers across the different FLC mutant organoids. Images are representative of similar results obtained within 3 lines per genotype. The box highlights the presence of "mixed phenotype" organoids, consisting of an organoid presenting with one half ALB+ and the other half CD44+. Scale bar = 50 μm.

Source data are provided as a Source Data file.

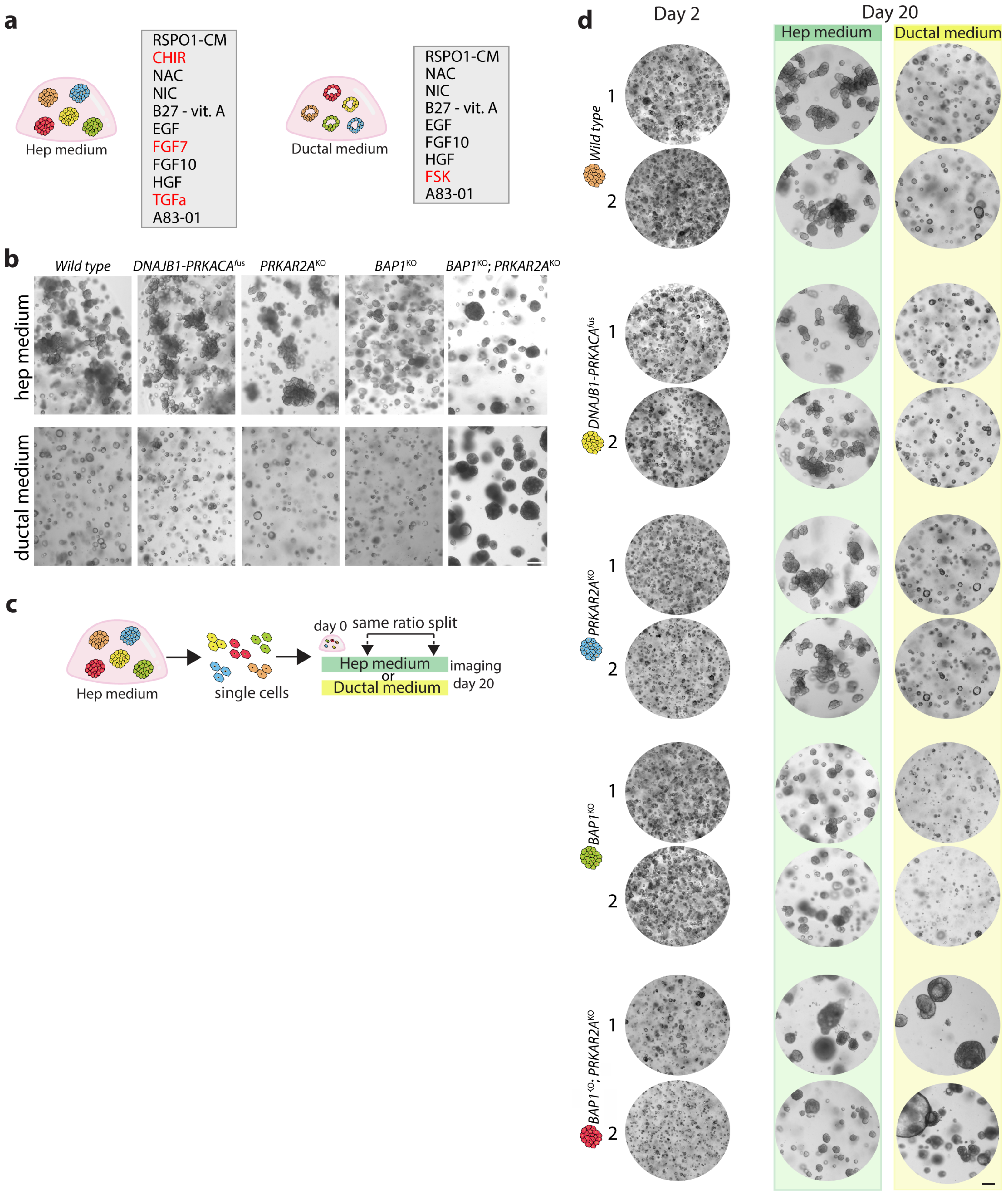
a
RSPO1-CM
CHIR
NAC
NIC
B27 - vit. A
EGF
FGF7
FGF10
HGF
TGFa
A83-01
Hep medium
RSPO1-CM
NAC
NIC
B27 - vit. A
EGF
FGF10
HGF
FSK
A83-01
Ductal medium
b
Wild type
DNAJB1-PRKACAfus
PRKAR2AKO
BAP1KO
BAP1KO; PRKAR2AKO
hep medium
ductal medium
c
Hep medium
single cells
day 0
same ratio split
Hep medium
or
Ductal medium
imaging
day 20
d
Day 2
Day 20
Hep medium
Ductal medium
Wild type
DNAJB1-PRKACAfus
PRKAR2AKO
BAP1KO
BAP1KO; PRKAR2AKO
1
2

Supplementary Figure 7. Growth behavior analysis of the FLC mutant organoid models upon culturing in hep or ductal medium.

a, Schematic overview comparing the medium composition of hep and ductal medium to stimulate hepatocyte and liver ductal cell organoid growth, respectively.

b, Representative brightfield images of the wild type and different FLC mutants when switched from hep medium to ductal medium as full-size organoids after 30 days. Images were taken ca. 30 days after switching the medium. Scale bar = 200 μm.

c, Schematic overview of testing the ability of the different FLC mutant and wild type organoids to grow in an environment stimulating liver ductal cell growth (ductal medium). Organoids are made into single cells and upon outgrowth thereafter split at the same ratio. Brightfield images are taken at the beginning (day 2) and end of the experiment (day 20).

d, Representative brightfield images of the wild type and different FLC mutants (2 independent lines per genotype are shown, indicated by the numbers) upon dissociation and at day 20 in hep or ductal medium upon 2 splitting events. Note that only the double mutant ($BAP1^{KO}$;$PRKAR2A^{KO}$) can grow in ductal medium. Scale bar = 75 μm.

b, d, Both representative of n = 3 independent experiments.

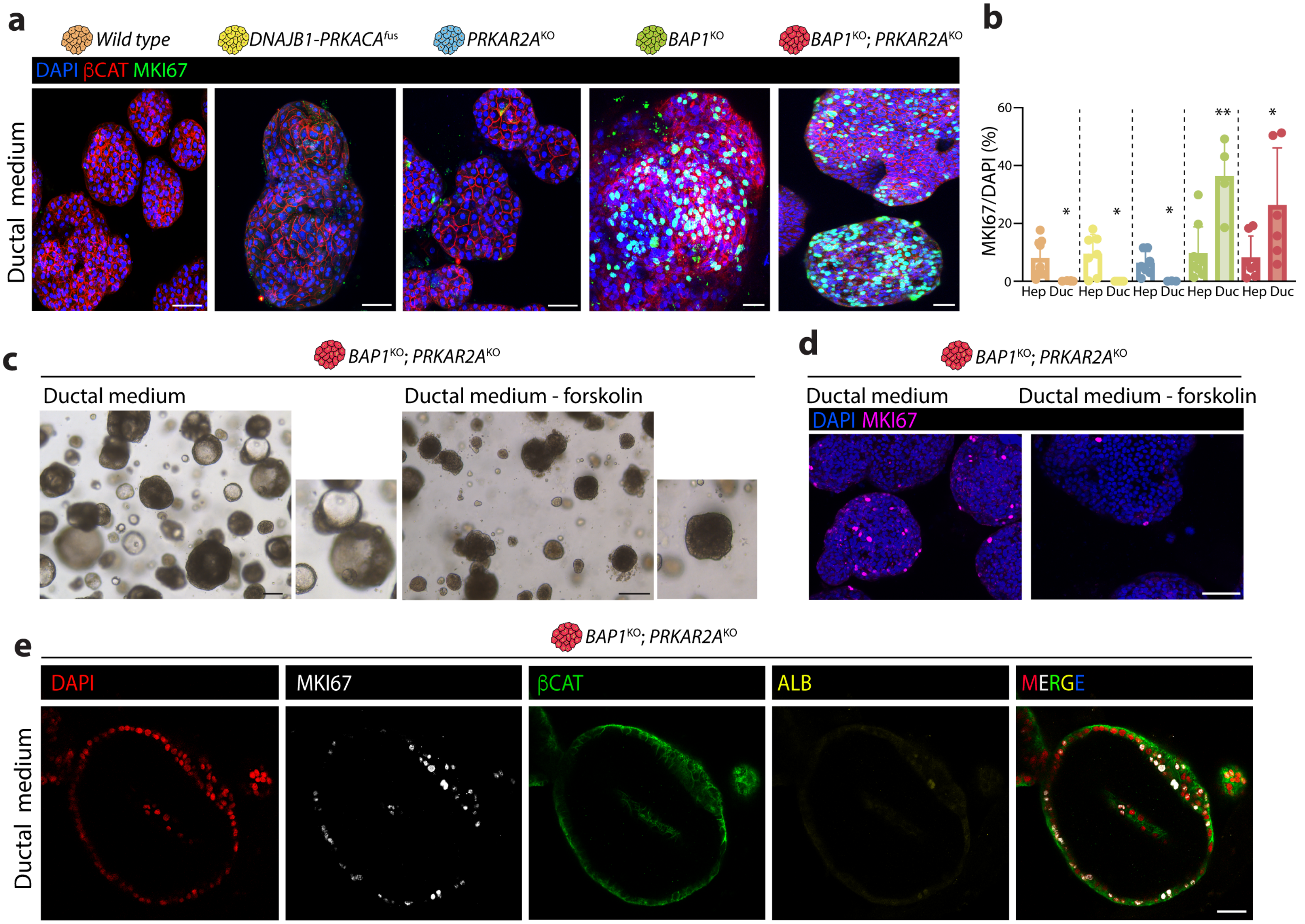

Supplementary Figure 8. Inclusion of a cAMP activator is essential for BAP1^{KO};PRKAR2A^{KO} organoid growth in ductal medium.

a, Representative images of MKI-67 staining with β-catenin staining of wild type organoids and the different FLC mutant organoids when cultured in ductal medium. Scale bar = 25 μm.

b, Quantification of the percentage of MKI67+ cells/DAPI+ cells within an organoid and comparison with the percentage of MKI67+ cells/DAPI+ cells when cultured in hep medium. Each dot represents quantification within an organoid. 2 independent lines per genotype were quantified. *$P < 0.05$, **$P < 0.01$ versus hep medium; two-tailed Student's t-test. Exact sample size and exact P values are provided in the source data.

c, Representative brightfield of BAP1^{KO};PRKAR2A^{KO} organoids grown in ductal medium with or without forskolin. Note the arrested growth in medium lacking forskolin. Scale bar = 200 μm.

d, Representative images of MKI-67 staining of BAP1^{KO};PRKAR2A^{KO} organoids grown in ductal medium with or without forskolin. Scale bar = 100 μm.

e, Representative section of a BAP1^{KO};PRKAR2A^{KO} organoid cultured in ductal medium stained for MKI-67, B-CAT and ALB, demonstrating the adoption of a cystic ductular-like organization where cells are monolayered. Note also the complete loss of ALB+ cells. Scale bar = 50 μm.

a-e, All representative of n = 2 independent experiments.

Source data are provided as a Source Data file.

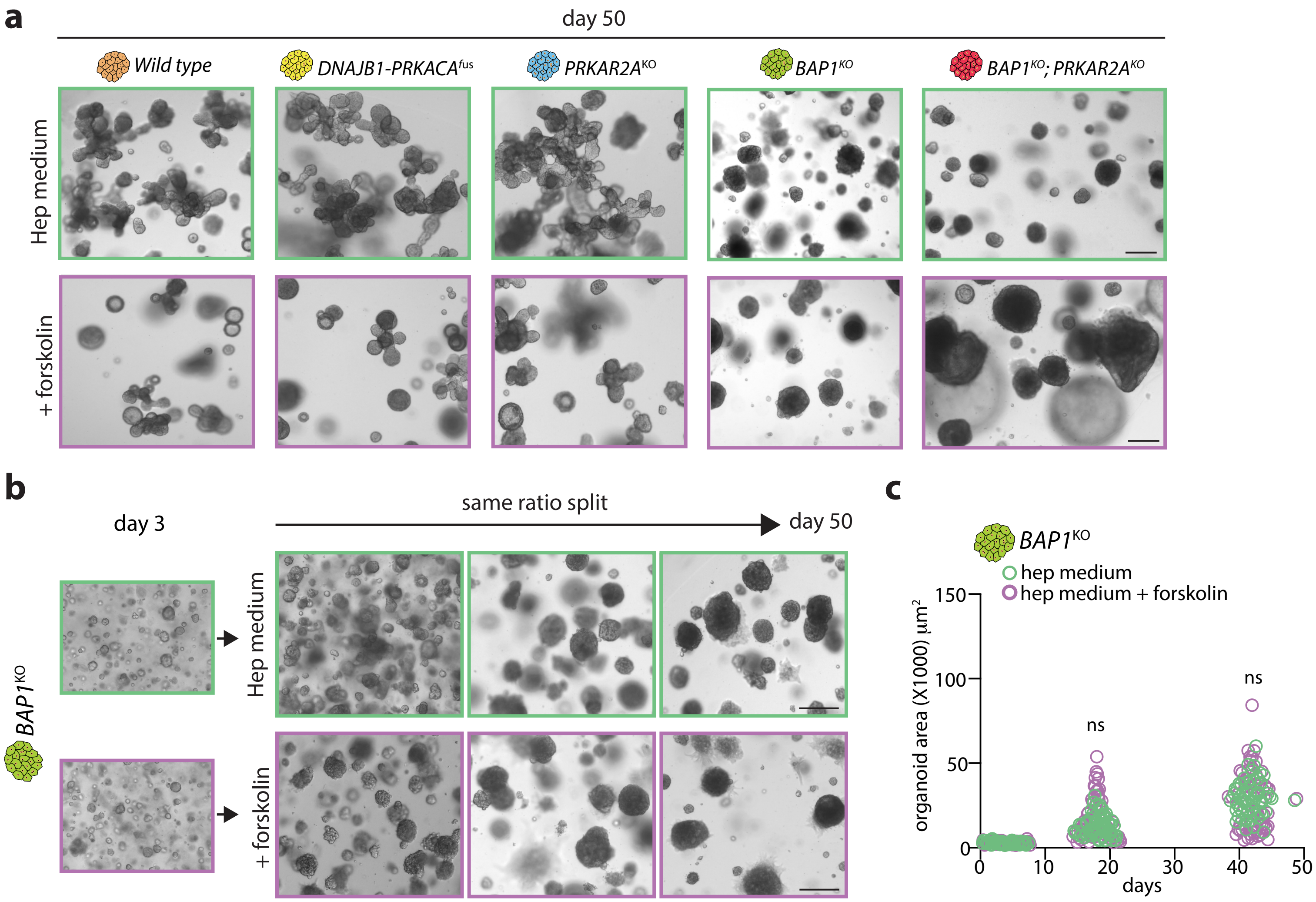

Supplementary Figure 9. Characterization of single BAP1-mutant hepatocytes cultured in hep medium supplemented with forskolin.

a, Representative brightfield images of wild type and the different FLC mutant organoids cultured (and split) for 50 days in hep medium or hep medium supplemented with forskolin. Scale bar = 200 μm.

b, Representative brightfield images of $BAP1^{KO}$ organoids cultured and split in hep medium or hep medium supplemented with forskolin over a 50-day time course. Scale bar = 200 μm.

c, Quantification of the organoid area of $BAP1^{KO}$ organoids when cultured in hep medium or hep medium supplemented with forskolin. Each dot represents the area of an individual organoid. Organoids from 3 independent lines per genotype were quantified. $^{**}P < 0.01$, $^{***}P < 0.001$ versus hep medium; two-tailed Student's t-test.

a-b, Both representative of n = 3 independent lines per genotype.

Source data are provided as a Source Data file.

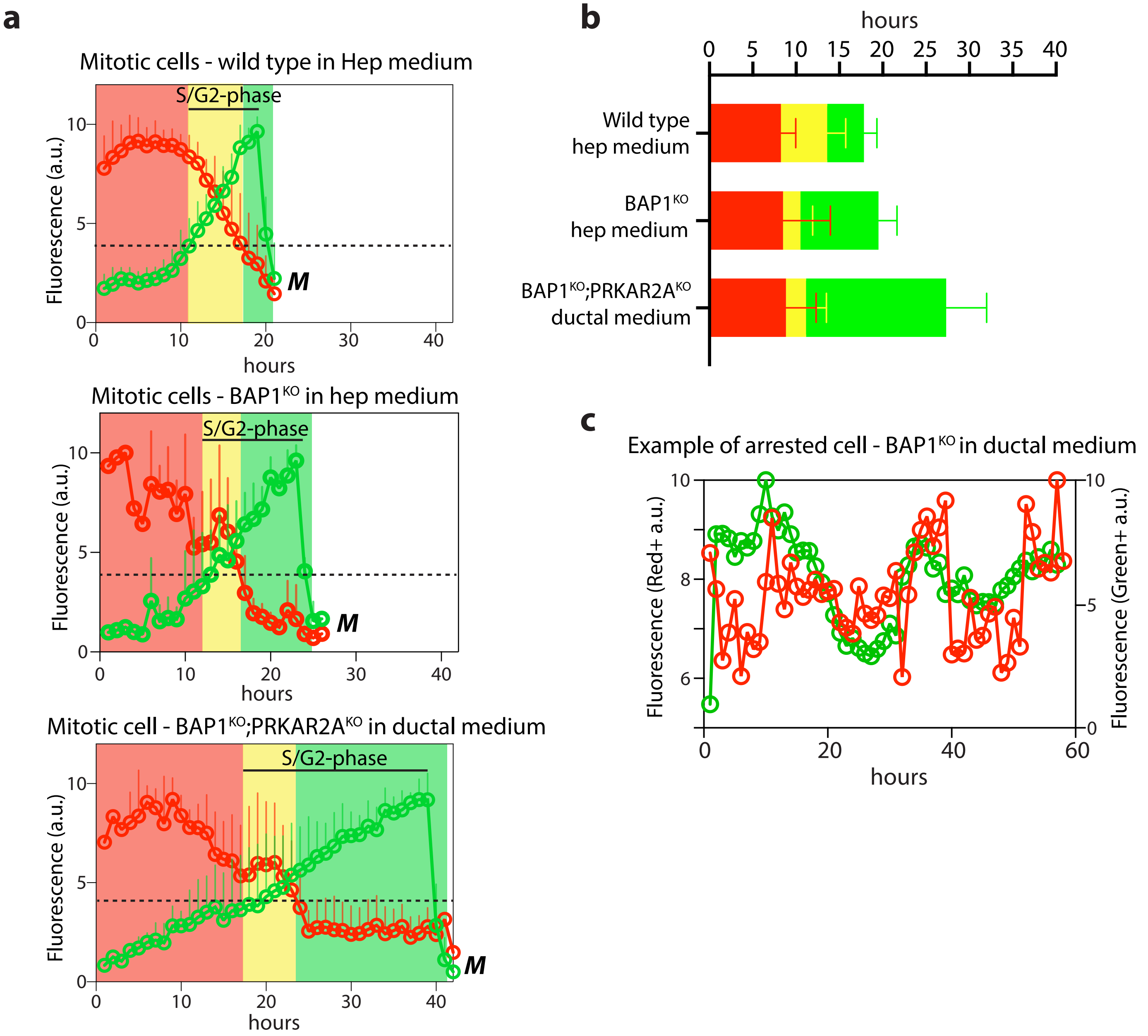

Supplementary Figure 10. FUCCI analysis of cell cycle behavior of BAP1-mutant cells with or without PRKAR2A loss.

a, Cell cycle behavior of mitotic cells of wild type organoids and BAP1^{KO} organoids cultured in hep medium and BAP1^{KO};PRKAR2A^{KO} organoids cultured in ductal medium. Mean ± SD is plotted. n = 12, 4 and 6 cells for wild type in hep medium, BAP1^{KO} in hep medium and BAP1^{KO};PRKAR2A^{KO} in ductal medium, respectively.

b, Cumulative plot showing the cell cycle length of mitotic cells of wild type organoids and BAP1^{KO} organoids cultured in hep medium and BAP1^{KO};PRKAR2A^{KO} organoids cultured in ductal medium, demonstrating the increased cell cycle length of BAP1^{KO};PRKAR2A^{KO} organoids. Mean ± SD is plotted. Sample size as in **b**.

c, Example of aberrant cell cycle behavior resulting in cell cycle arrest of a cell of a BAP1^{KO} organoid cultured in ductal medium.

a-c, All representative of n = 3 independent experiments.

Source data are provided as a Source Data file.

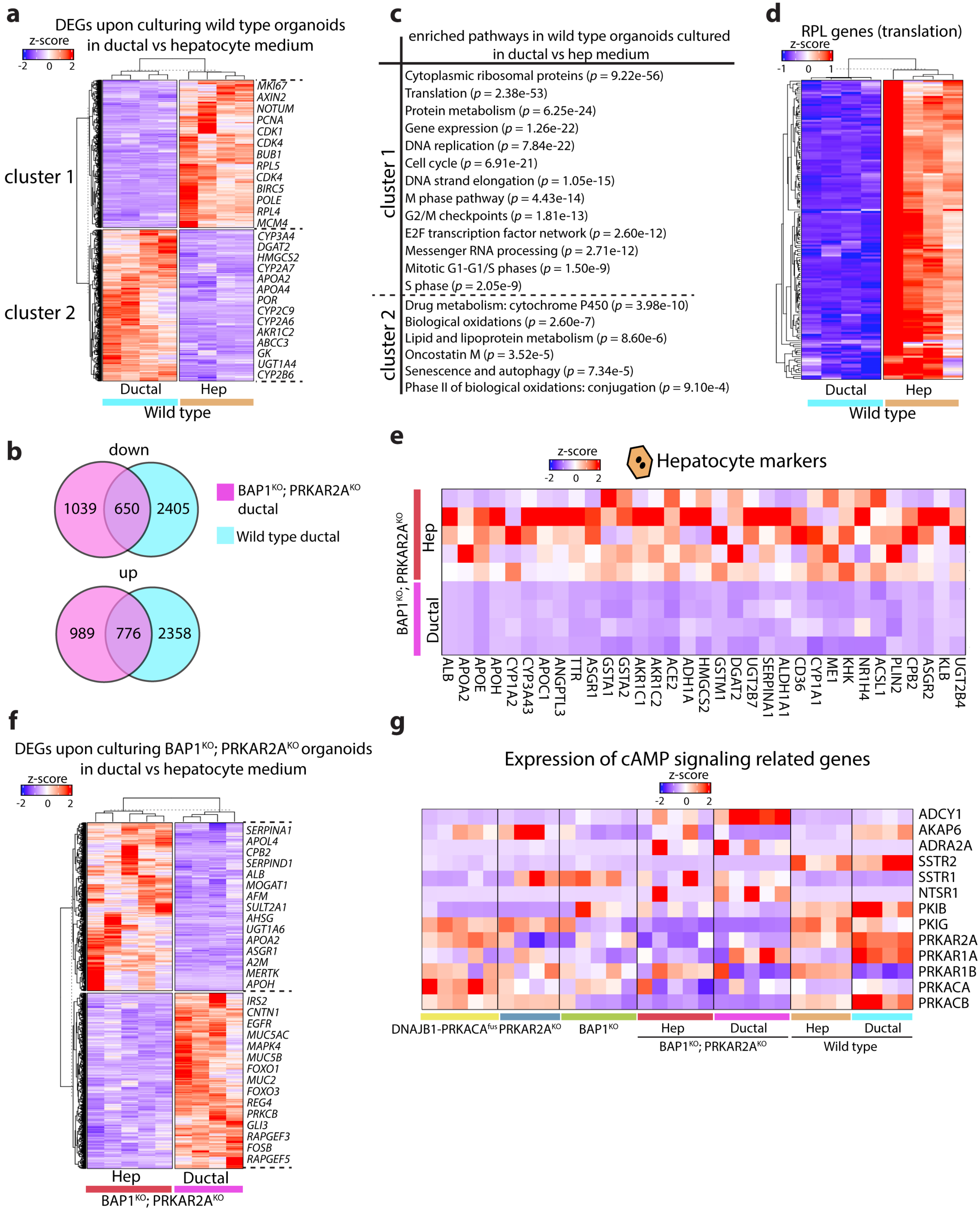
a
DEGs upon culturing wild type organoids in ductal vs hepatocyte medium
z-score
-2 0 2
cluster 1
cluster 2
MKI67
AXIN2
NOTUM
PCNA
CDK1
CDK4
BUB1
RPL5
CDK4
BIRC5
POLE
RPL4
MCM4
CYP3A4
DGAT2
HMGCS2
CYP2A7
APOA2
APOA4
POR
CYP2C9
CYP2A6
AKR1C2
ABCC3
GK
UGT1A4
CYP2B6
Ductal
Hep
Wild type
b
down
1039
650
2405
up
989
776
2358
BAP1KO; PRKAR2AKO ductal
Wild type ductal
c
enriched pathways in wild type organoids cultured in ductal vs hep medium
cluster 1
Cytoplasmic ribosomal proteins (p = 9.22e-56)
Translation (p = 2.38e-53)
Protein metabolism (p = 6.25e-24)
Gene expression (p = 1.26e-22)
DNA replication (p = 7.84e-22)
Cell cycle (p = 6.91e-21)
DNA strand elongation (p = 1.05e-15)
M phase pathway (p = 4.43e-14)
G2/M checkpoints (p = 1.81e-13)
E2F transcription factor network (p = 2.60e-12)
Messenger RNA processing (p = 2.71e-12)
Mitotic G1-G1/S phases (p = 1.50e-9)
S phase (p = 2.05e-9)
cluster 2
Drug metabolism: cytochrome P450 (p = 3.98e-10)
Biological oxidations (p = 2.60e-7)
Lipid and lipoprotein metabolism (p = 8.60e-6)
Oncostatin M (p = 3.52e-5)
Senescence and autophagy (p = 7.34e-5)
Phase II of biological oxidations: conjugation (p = 9.10e-4)
d
RPL genes (translation)
z-score
-1 0 1
Ductal
Hep
Wild type
e
z-score
-2 0 2
Hepatocyte markers
BAP1KO; PRKAR2AKO
Hep
Ductal
ALB
APOA2
APOE
APOH
CYP1A2
CYP3A43
APOC1
ANGPTL3
TTR
ASGR1
GSTA1
GSTA2
AKR1C1
AKR1C2
ACE2
ADH1A
HMGCS2
GSTM1
DGAT2
UGT2B7
SERPINA1
ALDH1A1
CD36
CYP1A1
ME1
KHK
NR1H4
ACSL1
PLIN2
CPB2
ASGR2
KLB
UGT2B4
f
DEGs upon culturing BAP1KO; PRKAR2AKO organoids in ductal vs hepatocyte medium
z-score
-2 0 2
SERPINA1
APOL4
CPB2
SERPIND1
ALB
MOGAT1
AFM
SULT2A1
AHSG
UGT1A6
APOA2
ASGR1
A2M
MERTK
APOH
IRS2
CNTN1
EGFR
MUC5AC
MAPK4
MUC5B
FOXO1
MUC2
FOXO3
REG4
PRKCB
GLI3
RAPGEF3
FOSB
RAPGEF5
Hep
Ductal
BAP1KO; PRKAR2AKO
g
Expression of cAMP signaling related genes
z-score
-2 0 2
ADCY1
AKAP6
ADRA2A
SSTR2
SSTR1
NTSR1
PKIB
PKIG
PRKAR2A
PRKAR1A
PRKAR1B
PRKACA
PRKACB
DNAJB1-PRKACAfus
PRKAR2AKO
BAP1KO
Hep
Ductal
BAP1KO; PRKAR2AKO
Hep
Ductal
Wild type

Supplementary Figure 11. Distinct transcriptomic rewiring of $BAP1^{KO}$;$PRKAR2A^{KO}$ organoids in ductal medium.
a, Heatmap depicting the expression of all differentially express genes found in wild type organoids cultured in ductal medium versus hep medium (|log2FC| > 0.5 and P-adj < 0.05, Wald test; DESeq2).
b, Venn diagrams showing the overlap between the differentially expressed genes (both up- and downregulated) detected upon culturing in ductal medium of wild type versus $BAP1^{KO}$;$PRKAR2A^{KO}$ organoids (|log2FC| > 0.5 and P-adj < 0.05, Wald test; DESeq2).
c, GO-term analysis of clusters 1 and 2 shown in a, showing the enriched pathways associated with the transcriptomic changes per cluster (Fisher exact test; Enrichr).
d, Heatmap depicting repressed expression of genes belonging to the RPL family found in wild type organoids when cultured in ductal versus hep medium (|log2FC| > 0.5 and P-adj < 0.05, Wald test; DESeq2).
e, Heatmap displaying the expression of selected hepatocyte markers in $BAP1^{KO}$;$PRKAR2A^{KO}$ organoids cultured in ductal or hep medium, demonstrating the enhanced repression upon culturing in ductal medium.
f, Heatmap depicting the expression of all differentially expressed genes found in $BAP1^{KO}$;$PRKAR2A^{KO}$ organoids cultured in ductal versus hep medium (|log2FC| > 0.5 and P-adj < 0.05, Wald test; DESeq2).
g, Heatmap displaying the expression of selected genes belonging to cAMP signaling in the different FLC mutant organoids as well as in wild type organoids in hep medium (and for wild type and $BAP1^{KO}$;$PRKAR2A^{KO}$ organoids also in ductal medium).
Exact P values for **a, b, d** and **f** are provided in **Supplementary Data 1**.
