# Supplementary material for: Organoid models of fibrolamellar carcinoma mutations reveal hepatocyte transdifferentiation through cooperative BAP1 and PRKAR2A loss
Source: Nat Commun. 2023 May 3;14:2377. doi: 10.1038/s41467-023-37951-6 (PMC10156813; doi:10.1038/s41467-023-37951-6)
Supplement: Supplementary file 2 — Description of Additional Supplementary Files [file 41467_2023_37951_MOESM2_ESM.pdf]

Description of Additional Supplementary Files

File Name: Supplementary Data 1
Description: Differentially expressed gene lists.

File Name: Supplementary Data 2
Description: Gene sets and cluster expression levels.

File Name: Supplementary Data 3
Description: Gene sets used for cell identity.

File Name: Supplementary Data 4
Description: Primers used in this study.
