# Supplementary material for: Organoid models of fibrolamellar carcinoma mutations reveal hepatocyte transdifferentiation through cooperative BAP1 and PRKAR2A loss
Source: Nat Commun. 2023 May 3;14:2377. doi: 10.1038/s41467-023-37951-6 (PMC10156813; doi:10.1038/s41467-023-37951-6)
Supplement: Supplementary file 7 — Reporting Summary [file 41467_2023_37951_MOESM7_ESM.pdf]

nature portfolio

Corresponding author(s): Delilah Hendriks
Benedetta Artegiani

Last updated by author(s): Mar 16, 2023

Reporting Summary

Nature Portfolio wishes to improve the reproducibility of the work that we publish. This form provides structure for consistency and transparency in reporting. For further information on Nature Portfolio policies, see our Editorial Policies and the Editorial Policy Checklist.

Statistics

For all statistical analyses, confirm that the following items are present in the figure legend, table legend, main text, or Methods section.

n/a	Confirmed	
☐	☒	The exact sample size (*n*) for each experimental group/condition, given as a discrete number and unit of measurement
☐	☒	A statement on whether measurements were taken from distinct samples or whether the same sample was measured repeatedly
☐	☒	The statistical test(s) used AND whether they are one- or two-sided<br>*Only common tests should be described solely by name; describe more complex techniques in the Methods section.*
☒	☐	A description of all covariates tested
☒	☐	A description of any assumptions or corrections, such as tests of normality and adjustment for multiple comparisons
☐	☒	A full description of the statistical parameters including central tendency (e.g. means) or other basic estimates (e.g. regression coefficient) AND variation (e.g. standard deviation) or associated estimates of uncertainty (e.g. confidence intervals)
☐	☒	For null hypothesis testing, the test statistic (e.g. *F*, *t*, *r*) with confidence intervals, effect sizes, degrees of freedom and *P* value noted<br>*Give P values as exact values whenever suitable.*
☒	☐	For Bayesian analysis, information on the choice of priors and Markov chain Monte Carlo settings
☒	☐	For hierarchical and complex designs, identification of the appropriate level for tests and full reporting of outcomes
☒	☐	Estimates of effect sizes (e.g. Cohen's *d*, Pearson's *r*), indicating how they were calculated

Our web collection on statistics for biologists contains articles on many of the points above.

Software and code

Policy information about availability of computer code

Data collection	Leica LAS X (v1.1)
Data analysis	ImageJ (v1.53), Photoshop CS4 (v24.1.1), Leica LAS X (v1.1), Prism 8.2.0, Rstudio (v2022.02.2), R (v4.1.0), DESeq2 (v3.16), factoextra (v1.0.7), GSEA (v.4.2.3), ggplot2 (v3.4.1), dendextend (v1.16.0), ComplexHeatmap (v3.16)

For manuscripts utilizing custom algorithms or software that are central to the research but not yet described in published literature, software must be made available to editors and reviewers. We strongly encourage code deposition in a community repository (e.g. GitHub). See the Nature Portfolio guidelines for submitting code & software for further information.

Data

Policy information about availability of data

All manuscripts must include a data availability statement. This statement should provide the following information, where applicable:

- Accession codes, unique identifiers, or web links for publicly available datasets
- A description of any restrictions on data availability
- For clinical datasets or third party data, please ensure that the statement adheres to our policy

Bulk RNA sequencing data generated in this study have been deposited in the GEO database under accession code GSE215785 (https://www.ncbi.nlm.nih.gov/geo/query/acc.cgi?acc=GSE215785). Source data are provided as a Source Data file.

Field-specific reporting

Please select the one below that is the best fit for your research. If you are not sure, read the appropriate sections before making your selection.

☒ Life sciences ☐ Behavioural & social sciences ☐ Ecological, evolutionary & environmental sciences

For a reference copy of the document with all sections, see nature.com/documents/nr-reporting-summary-flat.pdf

Life sciences study design

All studies must disclose on these points even when the disclosure is negative.

Sample size	No sample size calculation was performed to predetermine sample size. The sample sizes used were in teh same range of other studies in teh field. Results were validated across at least 3 different organoid lines per genotype.
Data exclusions	Based on whole transcriptome clustering, one DNAJB1-PRKACAfus sample clearly clustering apart from all other fusion organoids was defined as an outlier and excluded from further analysis, as further specified in the Methods.
Replication	Experiments were confirmed across multiple independent experiments and across multiple organoid lines and organoids per genotype. All attempts of replication were successful. Detailed information about replicate numbers are given in the figure legends and source data.
Randomization	Not applicable. There are no experiments that require randomization and therefore no randomization was performed.
Blinding	The researchers were not blinded since no experiments needed blinding for analysis.

Reporting for specific materials, systems and methods

We require information from authors about some types of materials, experimental systems and methods used in many studies. Here, indicate whether each material, system or method listed is relevant to your study. If you are not sure if a list item applies to your research, read the appropriate section before selecting a response.

Materials & experimental systems

n/a	Involved in the study	
☐	☒	Antibodies
☐	☒	Eukaryotic cell lines
☒	☐	Palaeontology and archaeology
☒	☐	Animals and other organisms
☐	☒	Human research participants
☒	☐	Clinical data
☒	☐	Dual use research of concern

Methods

n/a	Involved in the study	
☒	☐	ChIP-seq
☒	☐	Flow cytometry
☒	☐	MRI-based neuroimaging

Antibodies

Antibodies used	Anti-Ki67 #14-5698-82 (SolA15), Thermo Fisher dilution 1 to 1000; Phalloidin-Atto 647N Sigma-Aldrich #65906 dilution 1 to 1000; Anti-beta catenin (H-102) #sc-7199 Santa Cruz dilution 1 to 1000; Anti-ZO1 #PA5-19090 Thermo Fisher, dilution 1 to 500; Anti-ALB #A80-229A Thermo-Fisher dilution 1 to 300; Anti-cytokeratin #345779 BD bioscience Clone CAM5.2 dilution 1 to 500; Anti-cleaved-caspase3 (Asp175) #9661L Cell Signalling dilution 1 to 400; Anti-pCREB (Ser133) 9198S (87G3) Cell Signalling dilution 1 to 400; Anti-EMA ab15481 Abcam dilution 1 to 400; Anti-CD44 555476 Clone G44-26 BD Bioscience dilution 1 to 400; Anti-KRT7 Thermo Fisher MA5-11986 clone OV-TL 12/30 dilution 1 to 400, Anti-KRT19 13092S (D7F7W) Cell Signalling Technology dilution 1 to 500, Alexa-Fluor 488 anti-rabbit #A21206, Alexa-Fluor 647 anti-rat #A21247, Alexa-Fluor 488 anti-rabbit #A21206, Alexa-Fluor 568 anti-mouse #A10037, Alexa-Fluor 488 anti-goat #A11055.
Validation	All the antibodies were validated by the manufacturer and have been used across multiple publications.<br><br>Anti-Ki-67 purified (SolA15) #14-5698-82 (https://www.thermofisher.com/antibody/product/Ki-67-Antibody-clone-SolA15-Monoclonal/14-5698-82). This Antibody was verified by Cell treatment to ensure that the antibody binds to the antigen stated. Citations: e.g. PMID: 34525348, PMID: 34100459.<br>Phalloidin-Atto 647 #65906 (https://www.sigmaaldrich.com/NL/en/product/sigma/65906).<br>Citations; e.g. PMID32123335.<br>Anti-beta catenin #sc-7199 (https://www.scbt.com/p/beta-catenin-antibody-h-102) The use of this antibody has been validated in several publications e.g. PMID: 28793266, PMID: 25645929<br>Anti-ZO1 #PA5-19090 Thermo Fisher (https://www.thermofisher.com/antibody/product/PA5-19090.html?ef_id=Cj0KCQjw2cWgBhDYARIsALggUhqMYTVZX8hpXnjKbUTy6Ao3d8M31mNUy7Wz7lcVpQA-ymyyw60wHN4aAkZZEALw_wcB:G:s&s_kwcid=AL!3652!3!459737518508!!!g!!!10950825775!106531320406&cid=bid_pca_aup_r01_co_cp1359_pjt0000_bid00000_0se_gaw_dy_pur_con&gclid=Cj0KCQjw2cWgBhDYARIsALggU

hqMYTVZX8hpXnjKbUTy6Ao3d8M31mNUy7Wz7lcVpQA-ymyyw60wHN4aAkZZEALw_wcB), This Antibody was verified by Independent antibody to ensure that the antibody binds to the antigen stated and used in several publications e.g. PMID: 32403233.
Anti-ALB #A80-229A (https://www.thermofisher.com/antibody/product/Human-Albumin-Antibody-Polyclonal/A80-229A). By immunoelectrophoresis and ELISA this antibody reacts specifically with human albumin. Less than 0.1% cross reactivity to bovine, mouse and pig albumin was detected.
Anti-cytokeratin #345779 (https://www.bdbiosciences.com/en-eu/products/reagents/flow-cytometry-reagents/clinical-diagnostics/single-color-antibodies-asr-ivd-ce-ivd/anti-cytokeratin-purified.345779). Related citations PMID: 2579289, PMID: 3908491.
nti-cleaved-caspase3 (Asp175) #9661L Cell Signalling https://www.cellsignal.com/products/primary-antibodies/cleaved-caspase-3-asp175-antibody/9661). The use of this antibody has been validated in several previous publications e.g. PMID: 36280140, PMID: 36849492.
Anti-pCREB (Ser133) 9198S (https://www.cellsignal.com/products/primary-antibodies/phospho-creb-ser133-87g3-rabbit-mab/9198) This antibody has been validated using SimpleChIP® Enzymatic Chromatin IP Kits. Citations: e.g. PMID: 36443308
Anti-EMA ab15481 Abcam (https://www.abcam.com/products/primary-antibodies/muc1-antibody-ab15481.html). The use of this antibody has been validated in several previous publications e.g. PMID: 33247285, PMID: 32404936
Anti-CD44 555476 Clone G44-26 BD Bioscience https://www.bdbiosciences.com/en-eu/products/reagents/flow-cytometry-reagents/research-reagents/single-color-antibodies-ruo/purified-mouse-anti-human-cd44.555476) The use of this antibody has been validated in several previous publications e.g.PMID: 7515923
Anti-KRT7 https://www.thermofisher.com/antibody/product/Cytokeratin-7-Antibody-clone-OV-TL-12-30-Monoclonal/MA5-11986MA5-11986 clone OV-TL 12/30. This Antibody was verified by Relative expression to ensure that the antibody binds to the antigen stated.
Anti-KRT19 13092S (D7F7W) Cell Signalling Technology (https://www.cellsignal.com/products/primary-antibodies/keratin-19-antibody/3479). The use of this antibody has been validated in several previous publications, e.g. PMID: 33247284, PMID: 32123335

Eukaryotic cell lines

Policy information about cell lines

Cell line source(s)	The human hepatocyte organoid line used in this study was derived from human fetal liver tissue from abortion material from donors with informed consent under ethical permission (Leiden University Medical Center), as previously generated in Hendriks et al. Nat. Biotechnol. 2023 PMID: 36823355.
Authentication	The (CRISPR-engineered) organoid lines were not authenticated.
Mycoplasma contamination	All organoid lines were regularly assessed for mycoplasma contamination and scored negatively without exception.
Commonly misidentified lines (See ICLAC register)	N/A

Human research participants

Policy information about studies involving human research participants

Population characteristics	Anonymized healthy human fetal liver (GW 14) was used.
Recruitment	There was no pre-selection on the human fetal liver material used to generate organoids.
Ethics oversight	Anonymized human fetal liver became available after pregnancy terminations and upon informed consent and under ethical permission from the Leiden University Medical Center.

Note that full information on the approval of the study protocol must also be provided in the manuscript.
